# Metformin reduces the clonal fitness of *Dnmt3a*^R878H^ hematopoietic stem and progenitor cells by reversing their aberrant metabolic and epigenetic state

**DOI:** 10.21203/rs.3.rs-3874821/v1

**Published:** 2024-02-06

**Authors:** Mohsen Hosseini, Veronique Voisin, Ali Chegini, Angelica Varesi, Severine Cathelin, Dhanoop Manikoth Ayyathan, Alex C.H. Liu, Yitong Yang, Vivian Wang, Abdula Maher, Eric Grignano, Julie A. Reisz, Angelo D’Alessandro, Kira Young, Yiyan Wu, Martina Fiumara, Samuele Ferrari, Luigi Naldini, Federico Gaiti, Shraddha Pai, Aaron D. Schimmer, Gary D. Bader, John E. Dick, Stephanie Z. Xie, Jennifer J. Trowbridge, Steven M. Chan

**Affiliations:** 1 Princess Margaret Cancer Centre, Toronto, Ontario, Canada; 2 Donnelly Centre for Cellular and Biomolecular Research, Toronto, Ontario, Canada; 3 Department of Medical Biophysics, University of Toronto, Toronto, Ontario, Canada; 4 Department of Molecular Genetics, University of Toronto, Toronto, Ontario, Canada; 5 Department of Biochemistry and Molecular Genetics, University of Colorado Anschutz Medical Campus, Aurora, CO, USA; 6 The Jackson Laboratory, Bar Harbor, ME, USA; 7 San Raffaele Telethon Institute for Gene Therapy, IRCCS San Raffaele Scientific Institute, Milan, 20132, Italy.; 8 Vita-Salute San Raffaele University, Milan, 20132, Italy.; 9 Ontario Institute for Cancer Research, Toronto, Ontario, Canada

## Abstract

Clonal hematopoiesis (CH) arises when a hematopoietic stem cell (HSC) acquires a mutation that confers a competitive advantage over wild-type (WT) HSCs, resulting in its clonal expansion. Individuals with CH are at an increased risk of developing hematologic neoplasms and a range of age-related inflammatory illnesses^1–3^. Therapeutic interventions that suppress the expansion of mutant HSCs have the potential to prevent these CH-related illnesses; however, such interventions have not yet been identified. The most common CH driver mutations are in the DNA methyltransferase 3 alpha (*DNMT3A*) gene with arginine 882 (R882) being a mutation hotspot. Here we show that murine hematopoietic stem and progenitor cells (HSPCs) carrying the *Dnmt3a*^R878H/+^ mutation, which is equivalent to human *DNMT3A*^R882H/+^, have increased mitochondrial respiration compared with WT cells and are dependent on this metabolic reprogramming for their competitive advantage. Importantly, treatment with metformin, an oral anti-diabetic drug with inhibitory activity against complex I in the electron transport chain (ETC), reduced the fitness of *Dnmt3a*^R878H/+^ HSCs. Through a multi-omics approach, we discovered that metformin acts by enhancing the methylation potential in *Dnmt3a*^R878H/+^ HSPCs and reversing their aberrant DNA CpG methylation and histone H3K27 trimethylation (H3K27me3) profiles. Metformin also reduced the fitness of human *DNMT3A*^R882H^ HSPCs generated by prime editing. Our findings provide preclinical rationale for investigating metformin as a preventive intervention against illnesses associated with *DNMT3A*^R882^ mutation-driven CH in humans.

Mutations in *DNMT3A* are the most common genetic alterations in CH and are found in ~50–60% of CH carriers^2–5^. *DNMT3A* encodes a *de novo* DNA methyltransferase that catalyzes transfer of the methyl group from *S*-adenosylmethionine (SAM) to the C-5 position of cytosines in DNA, resulting in 5-methylcytosine (5mC) and production of *S*-adenosylhomocysteine (SAH). *DNMT3A* mutations are classified into those affecting the mutational hotspot at R882 and those affecting other parts of the gene (non-R882)^6^. Although both types of mutations are predicted to reduce methyltransferase activity, *DNMT3A*^R882^ mutations appear to confer a significantly higher risk of progression to acute myeloid leukemia (AML) compared with non-R882 *DNMT3A* mutations^7,8^. Thus, *DNMT3A*^R882^ mutations represent an important target for preventive intervention.

The mutations affecting R882 are almost invariably missense alterations and heterozygous^9,10^. *DNMT3A*^R882^ mutations have been shown to not only reduce the methyltransferase activity of the mutant protein but also decrease the activity of the WT protein in a dominant negative manner^10,11^. Consistent with these findings, the differentially methylated regions (DMRs) in human AML cells or peripheral blood cells harboring *DNMT3A*^R882^ mutations are predominantly hypomethylated compared with their WT counterparts^10,12^.

The impact of *DNMT3A* mutations on cell fate decisions of HSCs has previously been studied using genetically modified mouse models. In the *Dnmt3a*^R878H/+^ mouse model, the mutant HSCs are expanded and have a competitive advantage over WT HSCs^13^, thus recapitulating a key functional change associated with the mutation in humans. Here, we employed this model to identify differences in dependencies between *Dnmt3a*^R878H/+^ and WT HSPCs with the goal of targeting such dependencies to selectively suppress the expansion of mutant HSCs.

## *Dnmt3a*^R878H/+^ HSPCs exhibit increased mitochondrial respiration

**Fig. 1 |:**
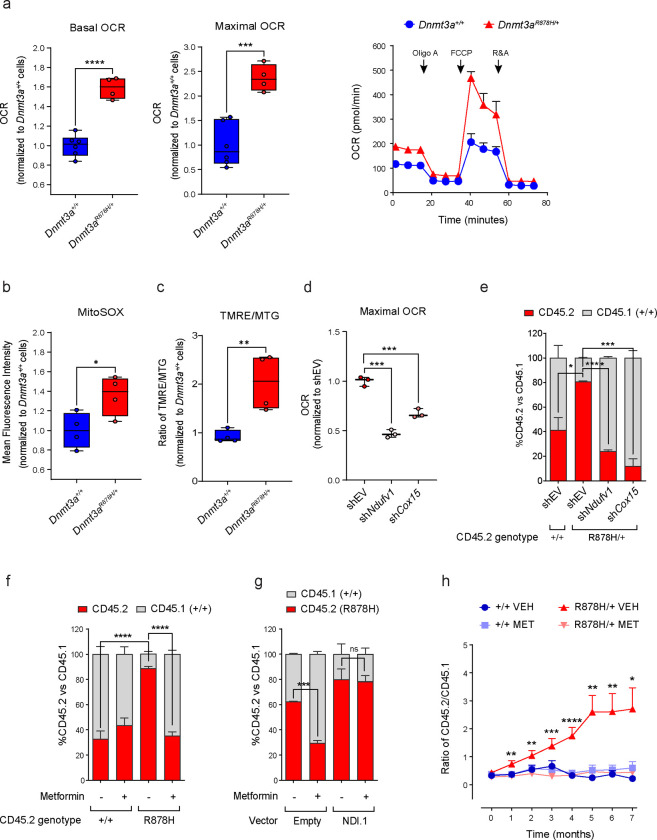
*Dnmt3a*^R878H/+^ HSPCs have increased mitochondrial respiration compared with *Dnmt3a*^+/+^ cells and are dependent on this metabolic reprogramming for their competitive advantage. **a,** Left panels, basal and maximal oxygen consumption rates (OCRs) in *Dnmt3a*^+/+^ and *Dnmt3a*^R878H/+^ LK HSPCs. Dots represent technical replicates. Right panel, OCRs of LK cells of the indicated genotype at baseline and at different time points following treatment with oligomycin A (Oligo A), FCCP, and rotenone plus antimycin A (R&A). n=4–6 technical replicates for each data point. Representative data of three independent experiments are shown. **b,** Mean fluorescence intensity of MitoSOX staining in *Dnmt3a*^+/+^ and *Dnmt3a*^R878H/+^ LK HSPCs. Dots represent samples from individual mice. **c,** Ratio of TMRE to MitoTracker Green (MTG) staining in *Dnmt3a*^+/+^ and *Dnmt3a*^R878H/+^ LK HSPCs. Dots represent samples from individual mice. **d,** Maximal OCR in *Dnmt3a*^R878H/+^ LK HSPCs transduced with an empty shRNA vector control (shEV) or a shRNA vector expressing sh*Ndufv1* or sh*Cox15*. Dots represent technical replicates. **e,** Proportion of CD45.2^+^ and CD45.1^+^ cells in a competition assay between CD45.2^+^ LK cells of the indicated genotype and CD45.1^+^ *Dnmt3a*^+/+^ LK cells. Both populations were transduced with the indicated shRNA vectors. n=3 technical replicates. **f,** Proportion of CD45.2^+^ and CD45.1^+^ cells in a competition assay between CD45.2^+^ LK cells of the indicated genotype and CD45.1^+^ *Dnmt3a*^+/+^ LK cells in the absence or presence of metformin at 50μM. n=3 technical replicates. Representative data of three independent experiments are shown. **g,** Proportion of CD45.2^+^ and CD45.1^+^ cells in a competition assay between CD45.2^+^ *Dnmt3a*^R878H/+^ LK cells and CD45.1^+^ *Dnmt3a*^+/+^ LK cells in the presence or absence of metformin at 50μM. The CD45.2^+^ *Dnmt3a*^R878H/+^ LK cells were transduced with an empty or NDI.1 overexpressing lentiviral vector. n=3 technical replicates. **h,** Ratio of CD45.2^+^ to CD45.1^+^ in peripheral blood cells collected from recipient mice at the indicated time points after starting treatment with metformin in the drinking water at 5g/L (MET) or no treatment (VEH). The mice were transplanted with CD45.1^+^ *Dnmt3a*^+/+^ bone marrow cells and CD45.2^+^ bone marrow cells of the indicated genotype 5 weeks prior to starting drug treatment. For months 0–4, data are from 3 independent experiments consisting of a total of 21–23 animals per condition. For months 5–7, data are from 1 experiment consisting of 6–7 animals per condition. Statistical significance was calculated in comparison with the untreated (VEH) arm of each genotype. In **a** (left panels), **b, c, d,** the box represents the interquartile range with the median indicated by the line inside the box. Whiskers extend to the minimum and maximum values. In **a** (right panel), **e, f, g, h,** data shown are mean ± SEM. Statistical significance (P values) was calculated using two-sided Student’s t-test with * P<0.05, ** P<0.01, *** P< 0.001, and *** P<0.0001. ns, not significant.

Analysis of publicly available RNA-sequencing (RNA-seq) datasets of primary AML samples revealed an increase in the expression of genes involved in oxidative phosphorylation (OXPHOS) in *DNMT3A*^R882^-mutated patient samples, but not *DNMT3A*^non-R882^-mutated samples, compared with *DNMT3A* WT samples (Extended Data Fig. 1a). These findings led us to explore if there are potential differences in mitochondrial function between *Dnmt3a*^R878H/+^ and *Dnmt3a*^+/+^ murine HSPCs. We found that *Dnmt3a*^R878H/+^ lineage negative, c-kit positive (LK) cells, which are enriched for HSPCs, possessed higher levels of basal and maximal oxygen consumption rates (OCRs) than *Dnmt3a*^+/+^ LK cells as determined by extracellular flux analysis (Fig. 1a). These differences were also observed in unfractionated whole bone marrow (WBM) cells, albeit by a smaller magnitude (Extended Data Fig. 1b). Furthermore, the level of mitochondrial reactive oxygen species (ROS) and ratio of mitochondrial transmembrane potential (ΔΨm) to mitochondrial mass (MM) were higher in mutant LK cells than in WT LK cells (Fig. 1b and 1c). Together, these findings indicate that the *Dnmt3a*^R878H^ mutation causes metabolic reprogramming in HSPCs resulting in upregulation of OXPHOS.

## Inhibition of mitochondrial respiration suppresses the competitive advantage of *Dnmt3a*^R878H/+^ HSPCs

We hypothesized that the enhanced mitochondrial respiration in *Dnmt3a*^R878H^ HSPCs is required for their competitive advantage over WT cells. To test this hypothesis, we first established an *in vitro* competition assay in which CD45.2^+^ *Dnmt3a*^R878H/+^ and CD45.1^+^ *Dnmt3a*^+/+^ LK cells were mixed at a ~2:3 ratio and cultured in cytokine-supplemented methylcellulose medium for ~10 days, followed by determination of the proportions of CD45.2^+^ and CD45.1^+^ cells (Extended Data Fig. 1c). A parallel competition assay between CD45.2^+^ *Dnmt3a*^+/+^ and CD45.1^+^ *Dnmt3a*^+/+^ LK cells mixed at the same starting ratio served as control. After the culture period, the proportion of CD45.2^+^ *Dnmt3a*^R878H/+^ cells was consistently ~20–30% higher than that of CD45.2^+^ *Dnmt3a*^+/+^ cells in the first passage and ~40–50% higher in the second passage (Extended Data Fig. 1d), demonstrating the competitive advantage of the mutant cells. Using this assay, we studied the impact of genetic knockdown of ETC subunits on the competitive advantage of *Dnmt3a*^R878H/+^ HSPCs by transducing the mixed CD45.2^+^ mutant/CD45.1^+^ WT population with lentiviral vectors expressing short-hairpin RNAs (shRNAs) against *Ndufv1* and *Cox15* (Extended Data Fig. 1e), which encode critical subunits in complex I and complex IV of the ETC, respectively. Downregulation of these genes reduced the maximal OCR and competitive advantage of CD45.2^+^ *Dnmt3a*^R878H/+^ cells (Fig. 1d and 1e), indicating that mutant HSPCs are dependent on OXPHOS to outcompete their WT counterparts.

To explore the translational relevance of this finding, we tested the impact of metformin, a commonly used oral anti-diabetic drug and pharmacologic inhibitor of Complex I^14^, on *Dnmt3a*^R878H/+^ LK HSPCs. Consistent with the genetic knockdown studies, treatment with metformin at a clinically relevant concentration (50 μM)^15^ suppressed the competitive advantage of mutant cells *in vitro* (Fig. 1f). This effect was rescued by expression of NDI1, a metformin-resistant yeast analog of Complex I^16,17^, thus confirming that metformin’s effect was due to on-target complex I inhibition (Fig. 1g). Metformin treatment also selectively reduced the clonogenic potential of *Dnmt3a*^R878H/+^ over *Dnmt3a*^+/+^ LK HSPCs in standard colony-forming unit (CFU) assays (Extended Data Fig. 1f).

To determine if the effect of metformin was relevant *in vivo* and over a longer treatment period, we conducted a competitive repopulation experiment by mixing CD45.2^+^ *Dnmt3a*^R878H/+^ or *Dnmt3a*^+/+^ WBM cells with CD45.1^+^ *Dnmt3a*^+/+^ WBM cells at a 2:3 ratio and transplanting the mixed cells into lethally irradiated recipients (Extended Data Fig. 1g). Five weeks after transplantation, the recipient mice were either left untreated or started on treatment with metformin in their drinking water at 5 mg/mL, a concentration that has previously been shown to result in blood concentrations comparable to those achievable in humans^18^. Peripheral blood (PB) chimerism analysis showed a stable ratio of CD45.2^+^ to CD45.1^+^ cells in mice that received CD45.2^+^ *Dnmt3a*^+/+^ control cells and the ratio was not affected by metformin treatment (Fig. 1h). In contrast, the ratio of CD45.2^+^ to CD45.1^+^ cells steadily increased over a 7-month period in mice that received CD45.2^+^ *Dnmt3a*^R878H/+^ cells, reflecting their competitive advantage over CD45.1^+^ WT cells (Fig. 1h). Importantly, metformin abrogated this competitive advantage up to 7 months (Fig. 1h). This effect was observed in both the myeloid and lymphoid compartments (Extended Data Fig. 1h). Our findings collectively indicate that inhibition of mitochondrial respiration is a potential strategy for targeting *DNMT3A*^R882^ mutation-driven CH.

## Metformin suppresses the competitive advantage of *Dnmt3a*^R878H/+^ HSCs

**Fig. 2 |:**
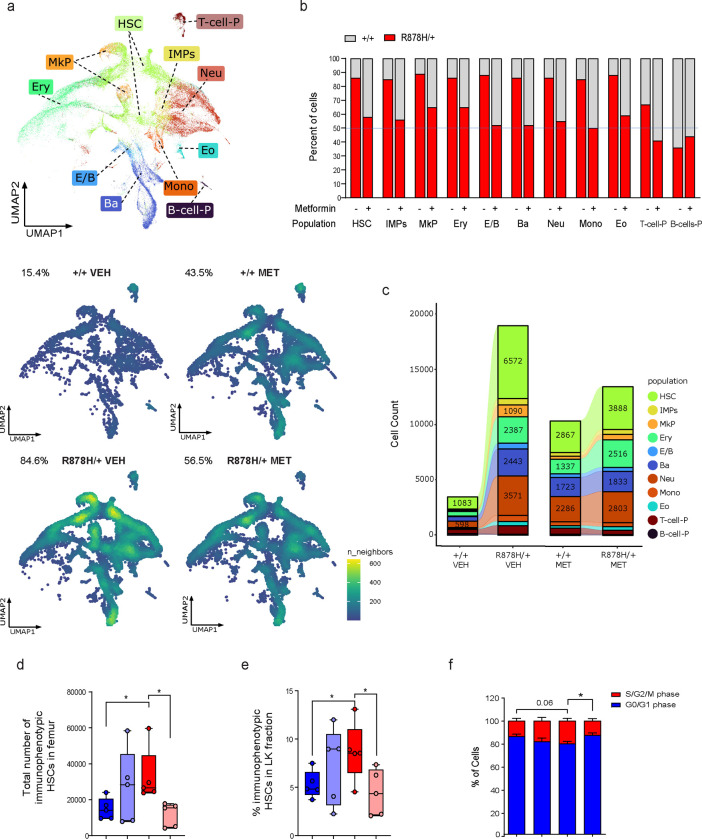
Metformin suppresses the competitive advantage of *Dnmt3a*^R878H/+^ HSCs. **a,** Top, dimensionality reduction using Uniform Manifold Approximation and Projection (UMAP) on all sequenced cells (n=46,225 cells). HSC = Hematopoietic stem cell; IMP = Immature myeloid progenitor; Mono = Monocyte progenitor, Neu = Neutrophil/granulocyte progenitor; E/B = Erythroid/basophil progenitor; Ery = Erythroid progenitor; MkP = Megakaryocyte progenitor; Ba = Basophil progenitor; Eo = Eosinophil progenitor; B-cell-P = B cell progenitor; T-cell-P = T cell progenitor. Bottom, UMAP cell density plots of CD45.1^+^ *Dnmt3a*^*+/+*^ cells vs. CD45.2^+^ *Dnmt3a*^R878H/+^ cells in LK-enriched BM samples collected from mice treated with vehicle (VEH) or metformin (MET). **b,** Proportion of CD45.1^+^ *Dnmt3a*^*+/+*^ cells vs. CD45.2^+^ *Dnmt3a*^R878H/+^ cells in each HSPC subset from untreated and metformin-treated LK samples. **c,** Sankey diagrams showing the absolute number of sequenced cells in each HSPC subset among CD45.1^+^ *Dnmt3a*^*+/+*^ vs. CD45.2^+^ *Dnmt3a*^R878H/+^ fractions in LK-enriched BM samples collected from mice treated with vehicle (VEH) or metformin (MET). **d,** Number of immunophenotypic HSCs (Lin^−^, c-Kit^+^, Sca-1^+^, CD150^+^, CD48^−^) in the right femur from mice transplanted with WBM cells of the indicated genotype and treated with or without metformin for 1 month. Dots represent samples from individual mice. **e,** Proportion of immunophenotypic HSCs in the LK fraction collected from mice transplanted with WBM cells of the indicated genotype and treated with or without metformin for 1 month. Dots represent samples from individual mice. **f,** Proportion of immunophenotypic HSCs in S/G2/M phase versus G0/G1 phase. Cells were collected from mice transplanted with WBM cells of the indicated genotype and treated with or without metformin for 1 month. n=5 mice per condition. In **d, e,** the box represents the interquartile range with the median indicated by the line inside the box. Whiskers extend to the minimum and maximum values. In **f,** data shown are mean ± SEM. Statistical significance (P values) was calculated using two-sided Student’s t-test with * P<0.05, ** P<0.01, *** P< 0.001, and *** P<0.0001.

Metformin suppressed the long-term competitive advantage of *Dnmt3a*^R878H/+^ donor cells *in vivo* (Fig. 1h), reflective of an impact at the HSC level. To provide independent evidence for impact at the HSC level and gain insights into metformin’s mechanism of action, we performed single cell RNA-seq (scRNA-seq) analysis on LK-enriched bone marrow (BM) cells from the untreated and metformin-treated recipients at the end of the 7-month treatment period (Extended Data Fig. 1g). The cells were collected from the mice transplanted with CD45.2^+^ *Dnmt3a*^R878H/+^ and CD45.1^+^ *Dnmt3a*^+/+^ competitor cells and stained with antibody-oligonucleotide conjugates (AOCs) specific for CD45.2 or CD45.1 to identify their donor origin. A total of 22,407 cells from untreated control mice (n=2) were sequenced, 84.6% of which were CD45.2^+^ *Dnmt3a*^R878H/+^ cells (Fig. 2a). In comparison, a total of 23,818 cells from metformin-treated mice (n=2) were sequenced, and the proportion of CD45.2^+^ *Dnmt3a*^R878H/+^ cells was significantly less at 56.5% (p<0.0001 by chi-square test) (Fig. 2a). To determine which HSPC subsets were affected, we annotated each cell based on their correlation with reference murine HSPC gene sets^19^ and identified 11 different hematopoietic subsets (Fig. 2a). Metformin treatment reduced the ratio of CD45.2^+^ to CD45.1^+^ cells in the HSC cluster as well as myeloid progenitor subsets (Fig. 2b and 2c).

To corroborate these findings, we transplanted *Dnmt3a*^R878H/+^ or *Dnmt3a*^+/+^ donor WBM cells from sex-matched littermates in a non-competitive manner into lethally irradiated recipients. Five weeks after transplantation, the recipients were either left untreated or started on treatment with metformin for one month (Extended Data Fig. 2a). In untreated mice, the number of immunophenotypic HSCs (Lin^−^, c-Kit^+^, Sca-1^+^, CD150^+^, CD48^−^) per femur and the proportion of HSCs in the LK fraction were higher in *Dnmt3a*^R878H/+^ recipients compared with *Dnmt3a*^+/+^ recipients (Fig. 2d,e). The expansion of mutant HSCs was associated with a trend towards a higher proportion of HSCs in S/G2/M phase (Fig. 2f). Metformin treatment reduced all these parameters in *Dnmt3a*^R878H/+^ recipients to levels comparable to those of untreated *Dnmt3a*^+/+^ recipients (Fig. 2d–f). Our findings demonstrate that metformin treatment suppresses the competitive advantage of *Dnmt3a*^R878H/+^ HSCs.

## Metformin acts by increasing the methylation capacity of *Dnmt3a*^R878H/+^ HSPCs

**Fig. 3 |:**
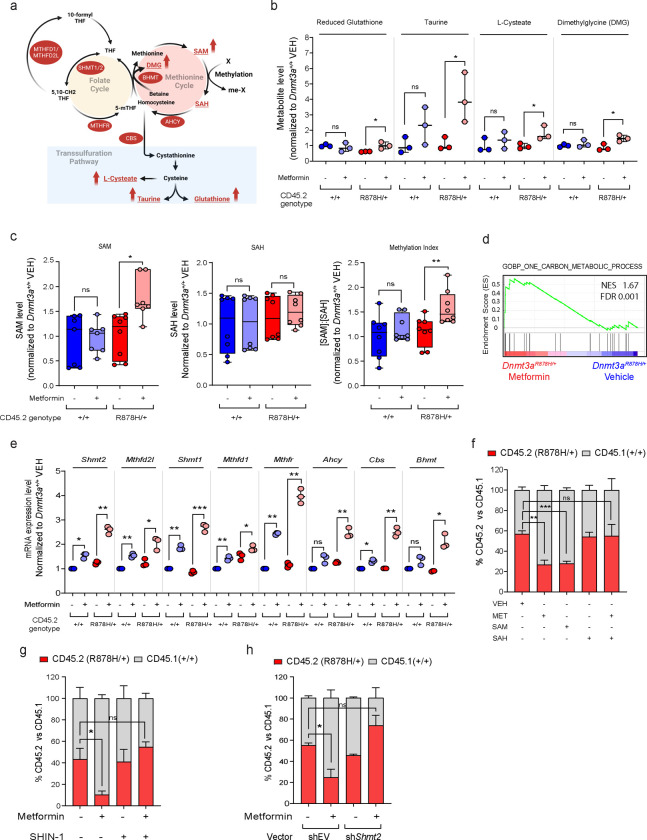
Metformin suppresses the fitness of *Dnmt3a*^R878H/+^ HSPCs by enhancing their methylation potential. **a,** Schematic diagram of the metabolic pathways involved in 1C metabolism. **b,** Levels of the indicated metabolites in LK cells isolated from mice transplanted with BM cells of the indicated genotype. The animals were either left untreated or treated with metformin for 1 month. Dots represent samples from individual mice. **c,** Levels of SAM and SAH and the ratio of [SAM]:[SAH] in LK cells isolated from mice transplanted with BM cells of the indicated genotype. The animals were either left untreated or treated with metformin for 1 month. Dots represent samples from individual mice. **d,** Gene set enrichment plot of bulk RNA-seq data comparing metformin-treated *Dnmt3a*^R878H/+^ LK cells (n=2 biological replicates) versus vehicle-treated *Dnmt3a*^R878H/+^ LK cells (n=2 biological replicates) using the indicated gene set (GO:0006730). **e,** Expression level of the indicated genes by RT-qPCR in LK cells isolated from mice transplanted with BM cells of the indicated genotype. The animals were either left untreated or treated with metformin for 1 month. Dots represent samples from individual mice. **f,** Proportion of CD45.2^+^ and CD45.1^+^ cells in a competition assay between CD45.2^+^ *Dnmt3a*^R878H/+^ LK cells and CD45.1^+^ *Dnmt3a*^+/+^ LK cells in the presence or absence of the indicated compounds. n=4 technical replicates. Representation data of 4 independent experiments are shown. **g,** Proportion of CD45.2^+^ and CD45.1^+^ cells in a competition assay between CD45.2^+^ *Dnmt3a*^R878H/+^ LK cells and CD45.1^+^ *Dnmt3a*^+/+^ LK cells in the presence or absence of the indicated compounds. n=4 technical replicates. Representation data from 3 independent experiments. **h,** Proportion of CD45.2^+^ and CD45.1^+^ cells in a competition assay between CD45.2^+^ *Dnmt3a*^R878H/+^ LK cells and CD45.1^+^ *Dnmt3a*^+/+^ LK cells in the presence or absence of metformin. Both populations were transduced with the indicated shRNA vectors. n=3 technical replicates. Representation data of 3 independent experiments are shown. In **b, c, e,** the box represents the interquartile range with the median indicated by the line inside the box. Whiskers extend to the minimum and maximum values. In **f, g, h,** data shown are mean ± SEM. Statistical significance (P values) was calculated using two-sided Student’s t-test for all comparisons except for **b** where one-sided Student’s t-test was used. * P<0.05, ** P<0.01, *** P< 0.001, and *** P<0.0001. ns, not significant.

Gene set enrichment analysis (GSEA) of the scRNA-seq data revealed an enrichment of genes associated with OXPHOS in *Dnmt3a*^R878H/+^ HSPCs relative to WT HSPCs and a decrease in expression of these genes with metformin treatment (Extended Data Fig. 3a). This unexpected result suggested that metformin could influence mitochondrial respiration not only through a direct inhibition of complex I but also through downregulation of OXPHOS-related genes. To further investigate its mechanism of action, we first studied the impact of *in vivo* metformin treatment on mitochondrial function of HSPCs. We performed extracellular flux analysis on freshly isolated LK-enriched BM cells from the animals that were untreated or treated with metformin for one month (Extended Data Fig. 2a). Metformin treatment reduced the basal and maximal OCRs as well as the ΔΨm of *Dnmt3a*^R878H/+^ LK cells to levels comparable to those of untreated *Dnmt3a*^+/+^ LK cells (Extended Data Fig. 3b and 3c). To uncover the impact of metformin on specific metabolic pathways, we performed a mass spectrometry-based metabolomic analysis of the untreated and treated LK cells of both *Dnmt3a* genotypes. This analysis, which focused on metabolites central to energy and redox metabolism, detected 101 named metabolites, of which 14 were significantly increased in metformin-treated mutant LK cells compared with untreated mutant cells (**Extended Data Table 1**). Intriguingly, 4 of the 14 upregulated metabolites (reduced glutathione (GSH), taurine, L-cysteate, and dimethylglycine) are involved in one-carbon (1C) metabolism through the methionine cycle (Fig. 3a and 3b). Since the methionine cycle generates SAM, these findings suggest that metformin could potentially affect SAM levels and the ratio of [SAM] to [SAH], which is also known as the methylation index, an indicator of cellular methylation potential. To test this hypothesis, we directly measured the intracellular concentrations of SAM and SAH, which were below the detection threshold of the bulk metabolomic analysis. Consistent with our hypothesis, the methylation index was higher in metformin-treated *Dnmt3a*^R878H/+^ LK cells than in untreated cells (Fig. 3c), indicative of an increase in their cellular methylation potential. Importantly, the impact of metformin on methylation index was observed only in *Dnmt3a*^R878H/+^ LK HSPCs but not in *Dnmt3a*^+/+^ LK cells (Fig. 3c).

To determine if the metformin-induced changes in methylation index could be due to alterations in the expression of genes involved in 1C metabolism, we performed bulk RNA-seq analysis of *Dnmt3a*^+/+^ and *Dnmt3a*^R878H/+^ LK-enriched cells that were untreated or treated with metformin for 1 month (Extended Data Fig. 2a). GSEA of the RNA-seq dataset showed that metformin treatment decreased the expression of genes associated with stemness and OXPHOS (Extended Data Fig. 3d,e), consistent with our earlier results. Importantly, it also revealed a significant enrichment of genes involved in 1C metabolism in *Dnmt3a*^R878H/+^ LK cells (Fig. 3d). To confirm these findings, we performed quantitative RT-PCR to measure the expression of 8 genes that encode enzymes in the folate and methionine cycles (*Shmt2*, *Mthfd2l*, *Shmt1*, *Mthfd1*, *Mthfr*, *Ahcy*, *Cbs*, *Bhmt*) and found that metformin treatment increased their expression in *Dnmt3a*^R878H/+^ LK cells (Fig. 3e). Metformin also upregulated expression of 6 of the 8 genes in *Dnmt3a*^+/+^ LK cells, but the magnitude of change was less (Fig. 3e). These findings suggest that metformin selectively increases the cellular methylation potential of mutant HSPCs by upregulating the expression of genes involved in 1C metabolism.

Based on the above findings, we hypothesized that metformin suppresses the clonal fitness of *Dnmt3a*^R878H/+^ cells by increasing their cellular methylation potential. To test this hypothesis, we investigated the impact of exogenous SAM and SAH on the competitive advantage of mutant LK cells using our *in vitro* assay (Extended Data Fig. 1c). The addition of exogenous SAM, which increases the methylation index, was sufficient to reduce the competitive advantage of *Dnmt3a*^R878H/+^ HSPCs (Fig. 3f). Conversely, exogenous SAH, which lowers the methylation index, counteracted the suppressive effect of metformin on mutant cells (Fig. 3f). To confirm these findings, we inhibited serine hydroxymethyltransferase 2 (SHMT2) activity as an alternative approach to lowering the [SAM]/[SAH] ratio. SHMT2 generates a one-carbon unit (5,10-methylenetetrahydrofolate) that is necessary for SAM synthesis through the folate and methionine cycles (Fig. 3a). In line with our hypothesis, both pharmacologic inhibition with SHIN-1, a potent SHMT inhibitor, and genetic knockdown of *Shmt2* expression rescued the suppressive effect of metformin on mutant HSPCs (Fig. 3g,h and Extended Data Fig. 3f). Altogether, these findings support a mechanism in which metformin selectively reduces the clonal fitness of *Dnmt3a*^R878H/+^ HSPCs by increasing their cellular methylation potential.

## Metformin reverses the aberrant DNA CpG methylation and H3K27me3 profiles in *Dnmt3a*^R878H/+^ HSPCs

**Fig. 4 |:**
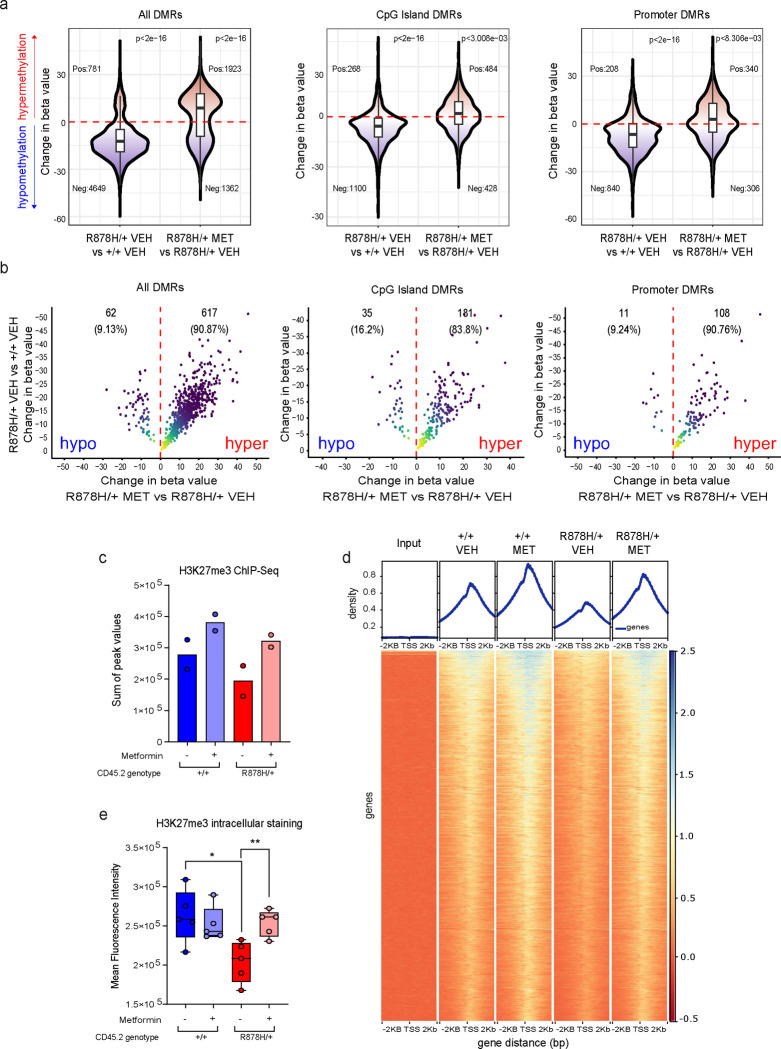
Metformin reverses the aberrant DNA CpG methylation and H3K27me3 profiles in *Dnmt3a*^R878H/+^ HSPCs. **a,** Violin plots of the difference in beta values at all differentially-methylated regions (DMRs), CpG island-associated DMRs, or promoter-associated DMRs in the comparison between untreated *Dnmt3a*^R878H/+^ LK samples versus untreated *Dnmt3a*^+/+^ LK samples (left) and between metformin-treated *Dnmt3a*^R878H/+^ LK samples versus untreated *Dnmt3a*^R878H/+^ LK samples (right). n=3 biological replicates for untreated *Dnmt3a*^+/+^ and metformin-treated *Dnmt3a*^R878H/+^ samples. n=4 biological replicates for metformin-treated *Dnmt3a*^+/+^ and untreated *Dnmt3a*^R878H/+^ samples. The P values adjacent to the plots were calculated using the one-sample Wilcoxon signed rank test determine if the median difference in beta values was significantly different from 0. **b,** Plot showing the change in beta values at overlapping DMRs between metformin-treated *Dnmt3a*^R878H/+^ samples versus untreated *Dnmt3a*^R878H/+^ samples on the X-axis and between untreated *Dnmt3a*^R878H/+^ samples versus untreated *Dnmt3a*^+/+^ samples on the Y-axis. **c**, Sum of peak values from H3K27me3 ChIP-seq analysis of LK HSPC samples collected from mice transplanted with bone marrow cells of the indicated genotype and treated with or without metformin for 1 month. Dots represent samples from individual mice. The means of the 2 biological replicates are shown. **d,** Distribution of H3K27me3 signals surrounding (±2KB) the transcription start site (TSS) regions with the highest signals (n=10,622) in the indicated samples. **e,** Mean fluorescent intensity of H3K27me3 staining by intracellular flow cytometry of LK HSPCs collected from mice transplanted with bone marrow cells of the indicated genotype and treated with or without metformin for 1 month. Dots represent samples from individual mice. In **e,** the box represents the interquartile range with the median indicated by the line inside the box. Whiskers extend to the minimum and maximum values. Statistical significance (P values) was calculated using two-sided Student’s t-test with * P<0.05, ** P<0.01, *** P< 0.001, and *** P<0.0001.

The mechanism by which *DNMT3A* mutations confer a fitness advantage to mutant HSCs is believed to be mediated through focal DNA hypomethylation secondary to reduced *de novo* DNA methylation activity^10,20^. In the context of *DNMT3A*^R882^ mutations where a WT copy of the gene remains, the residual DNA methylation activity is estimated to be ~20% of normal but is not absent^10^. We hypothesized that the metformin-induced increase in methylation index could augment this activity, resulting in a reversal of the aberrant DNA CpG hypomethylation pattern in mutant cells and consequent decrease in their fitness. A prediction based on this hypothesis is that a further reduction in residual DNMT3A activity should render the mutant cells resistant to the effect of metformin. In line with this prediction, we found that *Dnmt3a*^R878H/+^ LK cells expressing a *Dnmt3a* shRNA to knockdown residual DNMT3A activity maintained their competitive advantage over WT LK cells even in the presence of metformin *in vitro* (Extended Data Fig. 4a,b). Another prediction is that metformin treatment should increase the level of methylation at CpG sites that are differentially hypomethylated in *Dnmt3a*^R878H/+^ cells. To test this hypothesis, we performed reduced representation bisulfite sequencing (RRBS) analysis of LK-enriched BM cells from recipient mice that received *Dnmt3a*^R878H/+^ or *Dnmt3a*^+/+^ WBM cells from sex-matched littermate donors and were either untreated or treated with metformin for one month (Extended Data Fig. 2a). The RRBS technique, which enriches for CpG-rich regions, was chosen because DNMT3A preferentially catalyzes DNA methylation at CpG dinucleotides. We identified 5,430 DMRs in the comparison between untreated *Dnmt3a*^R878H/+^ (n=4) and untreated *Dnmt3a*^+/+^ (n=3) samples. Consistent with prior reports^10,20^, the majority of the DMRs (n=4,649; 85.6%) were hypomethylated in the untreated *Dnmt3a*^R878H/+^ samples (Fig. 4a). In the comparison between metformin-treated *Dnmt3a*^R878H/+^ (n=3) and untreated *Dnmt3a*^R878H/+^ (n=4) samples, we identified 3,285 DMRs, 1,923 (58.5%) of which were hypermethylated in the treated samples (Fig. 4a). We found 870 overlapping DMRs at the intersection between these two sets (Extended Data Fig. 4c). In line with our hypothesis, metformin treatment increased the methylation level at 617 (90.9%) of the 679 hypomethylated DMRs in *Dnmt3a*^R878H/+^ samples (Fig. 4b and Extended Data Fig. 4d). Similar findings were observed in the subsets of DMRs associated with CpG islands and gene promoter regions (Fig. 4a,b and Extended Data Fig. 4c,d). These findings demonstrate that metformin treatment can, at least in part, reverse the aberrant DNA CpG hypomethylation pattern in mutant cells.

In human CH, the *DNMT3A*^R882^ mutation has previously been reported to result in preferential DNA hypomethylation of targets of the polycomb repressive complex 2 (PRC2)^20^, which catalyzes the methylation of H3K27. In addition, the *Dnmt3a*^R878H^ mutation was previously found to be associated with a reduction in H3K27me3^21^, indicating its potential influence on another layer of epigenetic regulation. Given that PRC2-mediated methylation activity is also regulated by the ratio of [SAM]/[SAH], we hypothesized that metformin could reverse the aberrant H3K27 hypomethylation profile in *Dnmt3a*^R878H/+^ HSPCs. To test this hypothesis, we performed H3K27me3 chromatin immunoprecipitation followed by sequencing (ChIP-seq) analysis of *Dnmt3a*^R878H/+^ and *Dnmt3a*^+/+^ LK-enriched BM cells from the mice that were either untreated or treated with metformin for one month (Extended Data Fig. 2a). This analysis revealed a reduction in H3K27me3 levels globally and in the regions surrounding transcription start sites (TSSs) in the untreated *Dnmt3a*^R878H/+^ samples relative to the untreated *Dnmt3a*^+/+^ samples (Fig. 4c,d). Consistent with our hypothesis, metformin treatment restored H3K27me3 levels in *Dnmt3a*^R878H/+^ samples to a level comparable to that of untreated *Dnmt3a*^+/+^ samples (Fig. 4c,d). To confirm these findings using an orthogonal approach, we measured the level of H3K27me3 by intracellular flow cytometry. Similar to the ChIP-seq results, we found that *Dnmt3a*^R878H/+^ LK cells had lower levels of H3K27me3 than *Dnmt3a*^+/+^ LK cells and metformin treatment restored H3K27me3 levels in mutant cells to levels comparable to those of WT cells (Fig. 4e). Altogether, the above findings demonstrate that metformin treatment can reverse the aberrant epigenetic landscape in *Dnmt3a*^R878H/+^ HSPCs.

## Metformin decreases the fitness of human *DNMT3A*^R882H^ HSPCs

**Fig. 5 |:**
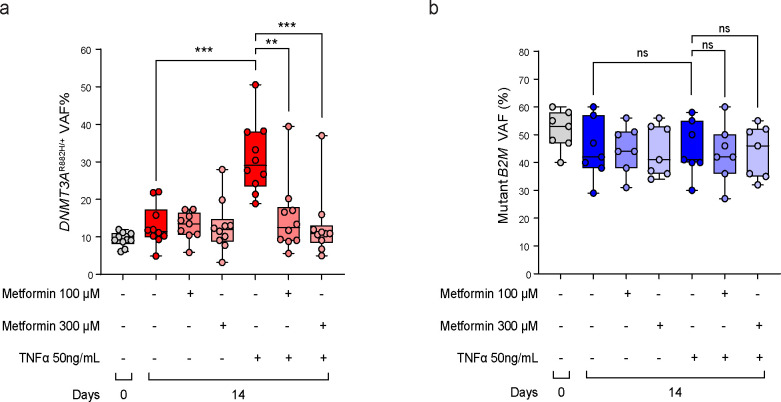
Metformin decreases the competitive advantage of human *DNMT3A*^R882H^ HSPCs. **a,** *DNMT3A*^R882H^ variant allele frequencies (VAFs) of prime edited human HSPCs at baseline (day 0) and after 14 days in culture in the presence or absence of TNFα or metformin. **b,** Mutant *B2M* VAFs of prime edited human HSPCs at baseline (day 0) and after 14 days in culture in the presence or absence of TNFα or metformin. Dots represent samples from individual cord blood donors. The box represents the interquartile range with the median indicated by the line inside the box. Whiskers extend to the minimum and maximum values. Statistical significance (P values) was calculated using two-sided Student’s t-test with * P<0.05, ** P<0.01, *** P< 0.001, and *** P<0.0001. ns, not significant.

To explore the relevance of our findings in human CH, we designed and optimized a new prime editing strategy to introduce the R882H mutation into the *DNMT3A* gene in purified CD34^+^ HSPCs from human cord blood (CB) samples as conventional homology-directed repair (HDR)-based CRISPR/Cas9 editing strategies are highly inefficient in human HSPCs^22^. The Cas nickase (nCas)-based prime editing technique has been shown to cause less cytotoxic/genotoxic stress and edit with higher precision and efficiency in long-term repopulating HSPCs^22,23^. Using the optimized prime editing strategy, we introduced the *DNMT3A*^R882H^ mutation in 10 HSPC samples from independent donors. As a negative control, we introduced a T>G single nucleotide variant (SNV) in exon 1 of the beta-2-microglobulin (*B2M*) gene which causes a premature stop codon^22^. The baseline mean variant allele frequency (VAF) on day 3 after prime editing was 9.3% for *DNMT3A*^R882H^ and 51.6% for the *B2M* SNV (Fig. 5a,b). On day 4, the edited cell pools were plated in methylcellulose medium to assess the relative fitness of the *DNMT3A*^R882H^ versus *DNMT3A*^WT^ cells in the presence or absence of tumor necrosis factor alpha (TNFα). The impact of TNFα, a proinflammatory cytokine, was studied because it has previously been shown to promote the competitive advantage of *Dnmt3a*^R878H/+^ HSCs^24^. After an additional 14 days in culture, the mean *DNMT3A*^R882H^ VAF remained stable in the absence of TNFα but increased to 30.9% in the presence of TNFα (Fig. 5a), indicative of a relative expansion of the mutant population in a proinflammatory milieu. Metformin treatment significantly prevented the expansion of *DNMT3A*-mutated cells in the presence of TNFα (Fig. 5a). Importantly, TNFα and metformin treatment did not affect the VAF of *B2M*-edited cells (Fig. 5b), indicating that the observed effects on *DNMT3A*^R882H^ HSPCs were not an artifact of prime editing. These results together support that metformin has the potential to suppress the fitness of *DNMT3A*^R882^-mutated clones in human CH upon inflammatory stress.

## Discussion

Targeting the cell intrinsic mechanisms critical for the selective advantage of mutant HSPCs in CH is a potential strategy for suppressing clonal expansion and lowering the risk of developing CH-related illnesses. Here, we found that upregulation of mitochondrial respiration is a key functional consequence of the *Dnmt3a*^R878H^ mutation and mutant HSPCs are dependent on this metabolic reprogramming to outcompete their WT counterparts. Importantly, this dependency was evident at the level of HSCs. Thus, our findings provide evidence that mitochondrial metabolism is a critical cell intrinsic regulator of clonal fitness in *DNMT3A*^R882^ mutation-driven CH. This notion is consistent with the growing body of evidence demonstrating a role for mitochondrial bioenergetics and dynamics in the regulation of stem cell fate.

Our discovery that *Dnmt3a*^R878H/+^ HSPCs are dependent on increased mitochondrial respiration has important therapeutic implications because many components of the ETC are druggable cellular targets. In this study, we focused on the therapeutic potential of metformin, a biguanide widely used in the treatment of diabetes. Although biguanides have been reported to target many cellular proteins, their inhibitory effect on complex I (NADH dehydrogenase) activity is the most well established and supported by structural evidence^14^. Indeed, our finding that ectopic expression of the metformin-resistant yeast analog of complex I (NDI1) rendered *Dnmt3a*^R878H/+^ HSPCs insensitive to effects of metformin strongly supports complex I as the main protein target. However, the observed reduction in mitochondrial respiration in metformin-treated *Dnmt3a*^R878H/+^ HSPCs was not due to complex I inhibition alone but also through the downstream downregulation of genes involved in OXPHOS. Results from our multi-omics studies suggest that metformin exerts its downstream effects on gene expression, at least in part, by increasing the methylation potential and consequently, augmenting the activity of DNMT3A, the PRC2 complex, and possibly other SAM-dependent methyltransferases in *Dnmt3a*^R878H/+^ HSPCs. This proposed mechanism is consistent with prior studies demonstrating an association between metformin exposure and an increase in 5mC and H3K27me3 levels in various cellular contexts^25–27^. It is noteworthy that metformin appears to preferentially increase the expression of genes involved in 1C metabolism and methylation index in *Dnmt3a*^R878H/+^ HSPCs over WT cells, indicating a degree of selectivity in its effects. Whether this selectivity is specific for metformin or common across other ETC inhibitors is unclear and warrants further investigations.

To explore the relevance of our findings in humans, we optimized a prime editing strategy to introduce the *DNMT3A*^R882H^ mutation into human HSPCs with high editing efficiencies. The prime editing technique has important advantages over Cas9 nuclease-based genome editing strategies that depend on the generation of DNA double-strand breaks (DSBs) which are highly toxic to HSCs. Although prime editing can still induce a small amount of DSBs, it is less genotoxic and can achieve high editing efficiencies in long-term repopulating HSPCs^22^. Our reported methodology represents an important technical resource for the study of *DNMT3A*^R882^ mutations in human HSPCs.

The presence of CH has been shown to be associated with an increased risk of developing not only hematologic malignancies but also a growing list of age-related illnesses. Interventions that effectively lower the risk of these adverse outcomes in CH carriers have the potential to positively impact the health of a large segment of the aging population. However, this goal is not yet possible due to the lack of known interventions that effectively suppress the expansion of mutant clones in CH. The ideal preventive intervention should not only be effective but also easy-to-administer and safe for long term use. Metformin fulfills these criteria and can readily be repurposed as a preventive treatment for *DNMT3A*^R882^-mutated CH carriers, especially those at high risk of malignant transformation or other CH-related illness. Our findings provide the preclinical rationale for studying this strategy in a prospective clinical trial.

## Materials and Methods

### Human cord blood samples

Cord blood (CB) CD34^+^ HSPCs were obtained with informed consent from Trillium Health, Credit Valley and William Osler Hospitals according to procedures approved by the University Health Network (UHN) Research Ethics Board. The mononuclear cells (MNC) were separated by centrifugation using Lymphoprep medium and then dissolved using ammonium chloride. The CD34^+^ cells were then isolated using the CD34 Microbead kit and purified using LS columns, following the directions provided by the manufacturer (Miltenyi). The CD34^+^ CB cells were preserved in a solution containing 50% PBS, 40% fetal bovine serum (FBS), and 10% DMSO at temperatures of −80°C or −150°C.

### Primary cell culture

CB CD34^+^ HSPCs were thawed via dropwise addition of X-VIVO^™^−10 media with 50% FBS and DNaseI (200 μg/ml). Cells were centrifuged at 350g for 10min at room temperature and seeded at the concentration of 5×10^5^ cells per ml in serum-free StemSpan medium (StemCell Technologies) supplemented with 2% glutamine, 100 ng/mL hSCF (R&D), 100 ng/mL hFlt3-L, 20 ng/mL hTPO, 1 μM SR1 (StemCellTechnologies) and 50nM UM171 (MedChemExpress LLC). Cells were cultured in a 5% CO_2_ humidified atmosphere at 37 °C.

### mRNA in vitro transcription

The PE3max plasmid was used to synthesize the mRNA encoding nCas9-RT through *in vitro* transcription, as described in Fuimara *et al* ^23^. Briefly, plasmid was linearized with SpeI (New England Biolabs) and purified by phenol-chloroform extraction. mRNAs were transcribed in vitro (5X MEGAscript T7 kit, Thermo Fisher), capped with 8 mM CleanCapAG (Trilink), purified (RNeasy Plus Mini Kit, Qiagen) and quality assessed by capillary electrophoresis (4200 TapeStation System, Agilent) ^23^. mRNAs were resuspended in RNase free water and stored at −80 °C.

### Gene editing of human HSPCs and analysis

A total of 1×10^5^–5×10^5^ cells were rinsed with PBS and subjected to electroporation using the P3 Primary Cell 4D-Nucleofector X Kit and Nucleofector 4D device, with program EO-100 from Lonza after 1 or 3 days of culture. The electroporation mixture consisted of 180 pM sgRNA from Synthego, 270 pM pegRNA from IDT, and 12μg PE3max mRNA. Following electroporation, cells were allowed to recuperate for 3 minutes at room temperature and subsequently maintained in culture according to prior findings. Three days following electroporation, CD34^+^ cells were harvested to obtain genomic gDNA for molecular investigation. The sgRNA sequences can be found in (Supplementary Excel Table 2). Standard pegRNA for the *B2M* positive control as described in Fuimara *et al* ^23^. Engineered pegRNA (epegRNA) targeting *DNMT3A* with a protective linker and motif at the 3’ end from guide degradation, were designed with pegFinder (http://pegfinder.sidichenlab.org/) and pegLIT (https://peglit.liugroup.us/)^28^.

### Human clonal competition assay

A clonal competition assay was conducted 24 hours after the editing procedure by placing 1000 cells per milliliter in a methylcellulose-based medium (MethoCultOptimum H4034, StemCell Technologies). The medium was supplemented with 10 ng/mL of hIL-6, and 10 ng/mL of hFlt3L. Each condition was subjected to four technical duplicates. After a period of two weeks following the plating process, the cells were collected and obtained for molecular analysis.

### Molecular analysis

The genomic DNA (gDNA) was extracted using the QIAamp DNA Micro Kit (Quiagen) from the pellet of in vitro cultured cells, or with QuickExtract (Epicentre) from cultivated cells in MethoCultOptimum H4034, following the instructions provided by the manufacturer. The efficiency of B2M PE was assessed using Sanger sequencing (performed by Eurofins Scientific) and the EditR software (available at http://baseeditr.com). To adapt EditR for B2M prime editing, we used as input the sequence TGGCCTTAGCTGTGCTCGC and selected the reverse complement orientation option as described in Fuimara *et al*^23^. The efficiency of DNMT3A R882H PE was assessed by ddPCR. QX200 Droplet Digital PCR System was used to examine 10–50 ng of gDNA for in vitro samples and 4 μl of gDNA for colonies in accordance with the manufacturer’s instructions. The VAF was calculated as the number of FAM-positive droplets divided by total droplets containing a target. The primers and probes are enumerated in (Supplementary Table 3).

### Mouse model and *in vivo* experiments

All *in vivo* experiments were performed in accordance with institutional guidelines approved by the University Health Network Animal care committee. C57BL/6J mice, also referred to as B6.CD45.2, and B6.SJL-Ptprca Pepcb/BoyJ mice, known as B6.CD45.1, were obtained from The Jackson Laboratory and held in the same facility for the duration of the study. The *Dnmt3a*fl-R878H/+ mice (JAX stock #032289) were crossbred with B6. CgTg(Mx1-cre)1Cgn/J mice (JAX stock #003556) and genotyped as described by Jackson Laboratory. All mice were female, and experiments initiated at 8–12 weeks of age. Mice were injected five times (once every other day) via intraperitoneal (IP) injection with 15 mg/kg high molecular weight polyinosinic-polycytidylic acid (pIpC) (Sigma-Aldrich ref: P1530) to induce Mx1-Cre recombinase expression. Before and after pIpC administration, genomic DNA was extracted from PB cells for recombination PCR. Primers used for genotyping are enumerated in (Supplementary Table 3). In addition, RNA was extracted, and cDNA synthesized from whole BM cells for Sanger sequencing to verify mutant allele expression.

CD45.2 *Dnmt3a* ^R878/+^ or *Dnmt3a* ^+/+^ BM cells (1E6 cells per mouse) were resuspended in Opti-MEM medium and transplanted by tail vein injection into 10 weeks old female CD45.2 Dnmt3a +/+ recipient mice conditioned with 12Gy of irradiation. For *in vivo* competition experiments, CD45.2 *Dnmt3a* ^R878/+^ or *Dnmt3a* ^+/+^ BM cells were mixed with CD45.1 *Dnmt3a* ^+/+^ BM cells from sex and age matched donor at a 1:2 ratio prior to transplantation. After five weeks following the transplantation, each group was subdivided before starting treatment with metformin at 5mg/ml in drinking water. Drinking water was replaced twice a week for the indicated period.

### Isolation of HSPCs from murine bone marrow

The bone marrow (BM) cells were enriched for Lin^−^Kit^+^ cells (HSPCs) using the EasySep mouse hematopoietic progenitor isolation kit (StemCell Technologies, Cat# 19856), followed by further enrichment using the c-KIT positive enrichment kit (StemCell Technologies, Cat# 18757).

### Murine *in vitro* competition assays

Competition experiments were conducted using 96-well flat-bottom tissue culture plates (Corning, Ref#351172). The bone marrow cells from 4-5-month-old mice were harvested after 5 weeks of post pIpC injection. HSPCs were enriched from BM cells as described above. The CD45.2 *Dnmt3a*^+/+^ HSPCs and CD45.2 *Dnmt3a*^R878H/+^ HSPCs (competing cells), were combined with CD45.1 *Dnmt3a*^+/+^ HSPCs in a proportion of 40% and 60%, respectively. The cell mixture was added to mouse MethoCultTM GF M3434 medium (StemCell Technologies, Cat # 3434) with a density of 200 cells per well, treatment was administered as indicated. Cells were incubated at 37°C with 5% CO2 for 10 days.

### Colony-forming unit (CFU) assays

A total of 3×10^3^ murine HSPC enriched cells were mixed with 1.1 mL of MethoCult^™^ GF M3434 medium, and metformin was administered at 100uM. Subsequently, the cell suspension was transferred to the wells of a 6-well tissue culture plate. After 10 days of culture, the colony formation was examined, with the potential to replate the cells as indicated.

### Flow cytometry

All flow cytometry analyses were conducted utilizing a Beckman Coulter CytoFLEX instrument, Cells were stained for 30 min at 4 °C with antibodies (listed in Supplementary Table 4) at the suggested dilutions in 100ul of FACS buffer (HBSS supplemented with 2% FBS and 0.1% sodium azide) and wash once prior to flow cytometry analysis. FCS files analysis was performed with FlowJo^™^ V10 software.

### Cell cycle assay

Murin enriched HSPC were stained for surface markers as described above prior to asses cell cycle via intracellular flow cytometry staining. The BD Cytofix/Cytoperm fixation and permeabilization reagent was utilized in accordance with the instructions provided by the manufacturer. Briefly, cells that had been fixed and permeabilized were stained for one hour at room temperature with a KI67 antibody diluted 1:200 in HBSS supplemented with 2% FBS and 0.1% sodium azide. The cells were subsequently rinsed in FACS buffer and subjected to staining with DAPI diluted 1:1,000 in PBS containing 1% FBS and 50mM EDTA at room temperature for 30 minutes. An IgG isotype antibody was tested for evaluating the level of isotype control staining.

### Quantification of mitochondrial reactive oxygen species (ROS) levels, mitochondrial membrane potential (ΔΨm), and mitochondrial mass

The levels of mitochondrial ROS, mitochondrial membrane potential (ΔΨm), and mitochondrial mass in freshly isolated mouse BM cells or HSPCs were assessed through flow cytometry utilizing MitoSOX^™^ Red reagent, tetramethylrhodamine ethyl ester (TMRE), and MitoTracker Green (MTG) probes (chemical reagents listed in Supplemental Table 5). The reagents were added directly to the cells in FACS buffer, resulting in final concentrations of 5 uM, 100 nM, and 200 nM, respectively. The cells were thereafter placed in an incubator set at a temperature of 37°C for 20 minutes. After the incubation period, the cells were washed with PBS 1X and then stained with Annexin V conjugated with Alexa Fluor 647 or Sytox^™^ Blue.

### Seahorse Analyzer-Mitostress test

All tests were carried out using the XF96 Extracellular Flux Analyzer from Seahorse Bioscience (North Billerica, MA). The sensor cartridge was hydrated overnight in a non-CO2 incubator using the calibration buffer medium supplied by Seahorse Biosciences (200 μl of buffer per well) on the day prior to the assay. The wells of Seahorse XFe96 microplates were coated the following day at 4°C with a 40 μl solution of Cell-Tak (Corning; Cat#354240) containing 22.4 μg/ml. The Cell-Tak-coated Seahorse microplate wells were subsequently rinsed with distilled water.

For cell plating, all cells were seeded at a density of 3*10^5^ cells per well on Seahorse XFe96 microplates, using XF base minimal DMEM media containing 11 mM glucose, 2 mM glutamine, and 1 mM sodium pyruvate. Following cell seeding, 180 μl of XF base minimal DMEM medium was added to each well, and the plate was centrifuged at 100 g for 5 minutes. Following a one-hour incubation of the seeded cells at 37°C in a non-CO2 incubator, the oxygen consumption rate (OCR) and extracellular acidification rate (ECAR) were evaluated under the baseline and in response to 1 μM Oligomycin, 1 μM carbonylcyanide-4-(trifluoromethoxy)-phenylhydrazone (FCCP), and 1 μM Antimycin and Rotenone (all from Sigma-Aldrich) using the XFe96 analyzer.

### RNA expression by RT-qPCR

mRNA was extracted from cells using Qiagen RNeasy plus kit and quantified on a Nanodrop spectrophotometer. Reverse transcription and quantitative PCR were performed at once using Luna^®^ Universal One-step RT-qPCR buffer and enzyme (NEB #E3005S). All qPCR experiments were done on a Bio-Rad CFX touch real-time PCR detection system.

### Bulk RNA Sequencing (RNA-seq)

RNA extraction from enriched murine HSPC was performed using the RNeasy Plus Mini Kit (QIAGEN, Cat #74136) following the instructions provided by the manufacturer. RNA samples were processed by Novogene Corporation in Sacramento, USA for sequencing analysis. Libraries were generated using the NEBNext Ultra RNA Library Prep Kit for Illumina, and sequencing was performed using the NovaSeq 6000 S4 with PE150 BP sequencing system. The readings were mapped to the mm10 reference genome using the STAR (v2.5) program. The HTSeq v0.6.1 software was utilized to tally the number of reads that were aligned to each individual gene.

### Cellular Indexing of Transcriptomes and Epitopes by Sequencing (CITE-seq):

We performed CITE-seq, a single-cell multi-omics technology that measures RNA and protein expression simultaneously in single cells. The dataset was derived from an in vivo competition experiment after 1 month of treatment, with 2 animals per treatment groups. Murin HSPC were enriched and then CD45.1 + cells (*Dnmt3a*^+/+^) and CD45.2 + cells (*Dnmt3a*^R879/+^) were marked using TotalSeq^™^-B antibodies (listed in Supplementary Table 4).

CITE-seq library generation was performed using 10x Genomics Chromium Single Cell 3′ v3.1_CellSurfaceProtein_RevD kit and the Novaseq 6000 sequencing system. 20000 cells for each of the 4 samples were sequenced. The scRNA reads were aligned against the mouse reference sequence mm10 using Cell Ranger (v6.1.2). The filtered Cell Ranger output was then used in the Seurat package v4 from Satija lab for further processing. Cells with more than 500 and less than 8000 number of genes per cells and less than 15% of mitochondrial genes were kept for the analysis. Fast integration using reciprocal PCA (RPCA) was used to find anchors across datasets to integrate the 4 samples. Normalization, variance stabilization and selection of 3000 top variable features of the molecular count data were performed using SCTransform followed by dimension reduction by PCA and UMAP embedding using the top 30 principal components. Hematopoietic subtypes were assigned to each cell using the AddModuleScore function and gene sets specific to murine hematopoietic populations as previously defined^19^. The top gene set enrichment score was establishing the selected annotation for each single cell. As additional filtering steps, the maturating erythroblastic cells expressing low level of Kit and Ptprc (CD45) were removed from the analysis and scDblFinder 1.16.0 was run on the Cell Ranger raw output of each individual sample to identify potential cell doublets. A total of 46225 cells were kept for downstream analyses.

The CD45.1 and CD45.2 sequencing antibody derived tags (ADTs) were log normalized. ADTs with a normalized value greater than 6 were identified as outlier points and removed from the analysis. Because the experiment is a competition assay, each sample contains a mix of cells expressing CD45.1 or CD45.2 at their surface, the ratio between CD45.1 and CD45.2 normalized ADTs was therefore used to label each cell as wild type (CD45.1> CD45.2) or mutant (CD45.1> CD45.2). To perform gene differential expression estimation, RNA counts from each defined hematopoietic cell population labelled as mutant and wildtype were transformed in a Single Cell Experiment object and aggregated for each of the metformin or vehicle samples using the sum of the counts in the R package scuttle (v1.0.4).

### Differential expression and Gene Set Enrichment Analysis (GSEA)

For both bulk RNA-seq and scRNA data, R package edgeR 3.36.0 was used to fit a generalized linear model to estimate differential expression between groups. All genes were ranked from the top up-regulated ones to down-regulated using the sign(logFC) * −log10(pvalue) formula. GSEA was performed using software from https://www.gsea-msigdb.org/gsea/index.jsp and ClusterProfiler 4.2.2 fgsea embedded functions using the rank gene files and 2 defined Gene Ontology Biological Process (GO BP) gene sets: oxidative phosphorylation (oxphos) (https://www.informatics.jax.org/go/term/GO:0006119) and 1-carbon metabolism (https://www.informatics.jax.org/vocab/gene_ontology/GO:0006730).

### Metabolomics analysis

Murine HSPCs were collected, washed with PBS, and pelleted by centrifugation. The cell pellets were then snap frozen and metabolites analyzed at the University of Colorado School of Medicine Metabolomics Core. Metabolites were extracted from frozen cells pellets at a concentration of 2×10^6^ cells/mL using a cold 5:3:2 methanol:acetonitrile:water solvent and quantified on a Thermo Vanquish UHPLC coupled to a Thermo Q Exactive mass spectrometer in a positive and negative ion modes (separate runs) exactly as described previously^29,30^. Signals were annotated and integrated using Maven in conjunction with the KEGG database and an in-house standard library as reported^31^.

### Measurement of *S*-adenosylmethionine and *S*-adenosylhomocysteine concentrations

The levels of *S*-adenosylmethionine (SAM) and *S*-adenosylhomocysteine (SAH) were quantified using the SAM and SAH Combo ELISA Kit developed by Cell Biolabs (Cat# STA-671-C). This enzyme immunoassay kit is specifically designed to accurately detect and measure SAH and SAM in cell lysate samples. In brief, for sample preparation, 3*10^6^ snap-frozen mouse HSPCs were thawed and sonicated in 1 mL of cold PBS 1X on ice. Following that, the homogenized samples underwent a 15-minute centrifugation at 10,000 g at 4°C. The supernatant obtained was carefully collected, kept on ice, and aliquoted for use in the assay. Any excess supernatant that was not utilized immediately was stored at a temperature of −80°C. The quantification of *S*-adenosylmethionine (SAM) and *S*-adenosylhomocysteine (SAH) concentrations was performed in accordance with the experimental protocols outlined in the manual provided by Cell Biolabs.

### Lentiviral production and transduction

Oligonucleotides used to generate shRNA vectors were chemically synthesized by Integrated DNA Technologies, annealed, and ligated into the BbsI site in the DECIPHER shRNA expression lentiviral vector (Cellecta; pRSI9-U6-(sh)-UbiC-TagRFP-2A-Puro) (Addgene plasmid# 28289). The TagRFP sequence has been replaced by the BFP sequence PCR -amplified and cloned into XbaI and BamHI restriction sites. The NDI1 coding sequence was PCR-amplified from PMXS-NDI1 (Addgene plasmid # 72876) and cloned into pLVX-EF1a-IRES-ZsGreen1 (Clontech, Cat # 631982) using EcoRI and SpeI restriction sites. The oligonucleotide and primers sequences are shown in (Supplementary Table 3).

For lentiviral production HEK293T cells were grown in RPMI 1640 medium (Wisent #350-035-CL) supplemented with 10% fetal bovine serum (FBS, Wisent #080–150) and 1% GlutaPlus (Wisent #609-066-EL). Cells were seeded into 150mm tissue culture plates at the density of 7 ×10^6^ cells/plate the day before transfection. On the day of transfection, cells were co-transfected with lentiviral vectors, psPAX2 (Addgene#12259), and pCMV-VSVG (Cell biolabs Part No.320023) plasmids using jetPRIME transfection reagents (Polyplus #CA89129–924) according to the manufacturer protocol. Viral particles were collected 48 and 72h post transfection and resuspended in HBSS (Gibco #14170112) +25mM HEPES (Thermo Fisher #15630–080). The combined supernatant was centrifuged at 450 × g and filtered through a 0.2 mm PES filter (Thermo Fisher Scientific, Cat # 564–0020). The filtered supernatant (40 mL) was mixed with 10 mL of PBS containing 20% (w/v) PEG 8000 (Sigma, Cat # 89510– 1KG-F), incubated overnight at 4°C, and centrifuged at 3,700 rpm for 30 mins at 4°C. The pellet containing lentiviral particles was resuspended in 2 mL of HBSS (Thermo Fisher Scientific, Cat # 14170–112) with 25 mM HEPES (Thermo Fisher Scientific, Cat # 15630–080), aliquoted, and store at −80°C.

For lentiviral transductions, non-TC-treated 24 well plates were coated with 20 mg/mL of Retronectin (Takara, Cat # T100B) for 2 hours at room temperature followed by aspiration and blocking with PBS containing 2% (w/v) BSA (Wisent Bioproducts, Cat # 800-096-EG) for 30 min at room temperature. After aspiration of the blocking buffer, the concentrated virus suspension was added to wells. The plates were then centrifuged at 3,700 rpm for two hours at 4°C to allow virus binding. Following centrifugation, unbound virus was aspirated, and 0.5 to 1×10^6^ AML cells were added. The plates were then transferred to a 37°C incubator to initiate lentiviral infection.

### Reduced-Representation Bisulfite Sequencing (RRBS) and analysis

Genomic DNA extracted from LK-enriched BM cell samples were submitted to Novogene (Sacramento, CA) for RRBS. Briefly, genomic DNA was digested with MspI, and the resulting fragments were end-repaired, A-tailed, and ligated with methylated adapters. After size-selection, bisulfite conversion was performed using the EZ DNA Methylation-Gold^™^ Kit (Zymo Research). The bisulfite-converted DNA was PCR-amplified to enrich for adapter-ligated fragments. RRBS libraries were quality-checked and sequenced on an Illumina HiSeq/NovaSeq platform, generating paired-end reads of 150bp nucleotides.

Raw data were trimmed to remove adapter and low-quality bases using Trimmomatic-0.36, followed by quality control assessment with FastQC (v0.11.5). Trimmed reads were aligned to mouse reference genome from Ensembl (GRCm38/mm10) and duplicated reads were removed. DNA methylation calls were extracted from the aligned reads as CpG coverage files. Differentially methylated regions (DMRs) were identified using the open-source R package methylKit (v1.26.0)^32,33^. CpG sites on unmapped genome assembly contigs were removed, and remaining CpG sites were filtered to exclude CpGs with <10× coverage PCA analysis in R. We used methylKit to perform pairwise comparisons to identify DMRs between untreated *Dnmt3a*^R878H/+^ vs. untreated *Dnmt3a*^+/+^ samples, and between metformin-treated *Dnmt3a*^R878H/+^ and untreated *Dnmt3a* ^R878H/+^ samples. To this end, the genome was tiled into 500bp non-overlapping bins. To calculate DMR p-values, a logistic regression test was used methylKit. P-values were adjusted for multiple testing (i.e., q-value) via the SLIM method^34^. DMRs with p-value < 0.01 were used for downstream analysis. CpG islands were annotated by using the University of California Santa Cruz (UCSC) (https://genome.ucsc.edu/index.html) database with using plyranges R package (v1.20.0)^35^. Promoters were defined as 1kb upstream and 150bp downstream around the transcription start site (TSS) and annotated by ChIPseeker R package (v1.36.0)^36,37^.

### H3K27me3 Chromatin-immunoprecipitation sequencing (ChIP-seq)

LK-enriched BM cells were fixed with 1% formaldehyde for 15 min according to the Active Motif ChIP cell fixation protocol. Fixed cell pellets were submitted to Active Motif (Carlsbad, CA) for ChIP-Seq. Briefly, 15ug chromatin and 4ul of antibody against H3K27me3 (Active Motif cat# 39155) were used to immunoprecipitated genomic DNA regions of interest. Illumina base-cell data were processed and demultiplexed using bcl2fastq2 v2.20 and low-quality bases with Phred scores less than 33 were trimmed. 75 bp single-end sequence reads were subsequently mapped to the genome through BWA v0.7.12 algorithm with default settings. Low quality reads were filtered out and PCR duplicates were removed. Aligned sequencing reads, or tags, were extended to 200bp from the 3’ end, followed by dividing the genome into 32bp bins and counting the number of fragments in each bin. The resulting histograms (genomic “signal maps”) were stored in bigWig files. Peak locations were determined using the MACS algorithm (v2.1.0) with a cutoff of p-value =1e-7. Peaks that were on the ENCODE blacklist of known false ChIP-Seq peaks were removed. 18556 peaks that were identified in at least one sample with a cutoff p-value of 1e-7 were all merged in a common matrix and the total number of present peaks as well as averaged peak values were calculated and plotted for each sample and condition using the R package ggplot2. A t-test was used to assess difference in mean between each condition. ChIP-Seq profiles +−2kb of the transcription start site (TSS) of 10,622 transcripts representing unique genes was created using the plotHeatmap function of DeepTools 3.5.1.

### Quantification and Statistical analysis

Statistical analyses were carried out according to the specifications detailed in the figure legends using GraphPad Prism v10 (GraphPad Software, La Jolla, CA). The figure legends contain details regarding the quantity of experimental repetitions or animals in each group. Statistical significance was defined as P values < 0.05. In the context of GSEA analysis, statistical significance was established using P values below 0.05 and FDR values below 0.05. This rigorous approach was employed to ascertain statistical significance in the GSEA results.

## Supplementary Material

Supplement 1

## Data Availability

Raw and processed data from the bulk RNA-seq, scRNA-seq, RRBS, and ChIP-seq experiments will be submitted to a publicly available repository (GEO).

## References

[1] Jaiswal S. Clonal Hematopoiesis and Risk of Atherosclerotic Cardiovascular Disease. N Engl J Med 377, 111–121, doi:10.1056/NEJMoa1701719 (2017). 28636844 PMC6717509

[2] Jaiswal S. Age-related clonal hematopoiesis associated with adverse outcomes. N Engl J Med 371, 2488–2498, doi:10.1056/NEJMoa1408617 (2014). 25426837 PMC4306669

[3] Genovese G. Clonal hematopoiesis and blood-cancer risk inferred from blood DNA sequence. N Engl J Med 371, 2477–2487, doi:10.1056/NEJMoa1409405 (2014). 25426838 PMC4290021

[4] Bick A. G. Inherited causes of clonal haematopoiesis in 97,691 whole genomes. Nature 586, 763–768, doi:10.1038/s41586-020-2819-2 (2020). 33057201 PMC7944936

[5] Buscarlet M. DNMT3A and TET2 dominate clonal hematopoiesis and demonstrate benign phenotypes and different genetic predispositions. Blood 130, 753–762, doi:10.1182/blood-2017-04-777029 (2017). 28655780

[6] Venugopal K., Feng Y., Shabashvili D. & Guryanova O. A. Alterations to DNMT3A in Hematologic Malignancies. Cancer Res 81, 254–263, doi:10.1158/0008-5472.CAN-20-3033 (2021). 33087320 PMC7855745

[7] Young A. L., Tong R. S., Birmann B. M. & Druley T. E. Clonal hematopoiesis and risk of acute myeloid leukemia. Haematologica 104, 2410–2417, doi:10.3324/haematol.2018.215269 (2019). 31004019 PMC6959179

[8] Jawad M. DNMT3A R882 Mutations Confer Unique Clinicopathologic Features in MDS Including a High Risk of AML Transformation. Front Oncol 12, 849376, doi:10.3389/fonc.2022.849376 (2022). 35296003 PMC8918526

[9] Larsson C. A., Cote G. & Quintas-Cardama A. The changing mutational landscape of acute myeloid leukemia and myelodysplastic syndrome. Mol Cancer Res 11, 815–827, doi:10.1158/1541-7786.MCR-12-0695 (2013). 23645565 PMC4123851

[10] Russler-Germain D. A. The R882H DNMT3A mutation associated with AML dominantly inhibits wild-type DNMT3A by blocking its ability to form active tetramers. Cancer Cell 25, 442–454, doi:10.1016/j.ccr.2014.02.010 (2014). 24656771 PMC4018976

[11] Kim S. J. A DNMT3A mutation common in AML exhibits dominant-negative effects in murine ES cells. Blood 122, 4086–4089, doi:10.1182/blood-2013-02-483487 (2013). 24167195 PMC3952368

[12] Smith A. M. Functional and epigenetic phenotypes of humans and mice with DNMT3A Overgrowth Syndrome. Nat Commun 12, 4549, doi:10.1038/s41467-021-24800-7 (2021). 34315901 PMC8316576

[13] Loberg M. A. Sequentially inducible mouse models reveal that Npm1 mutation causes malignant transformation of Dnmt3a-mutant clonal hematopoiesis. Leukemia 33, 1635–1649, doi:10.1038/s41375-018-0368-6 (2019). 30692594 PMC6609470

[14] Bridges H. R. Structural basis of mammalian respiratory complex I inhibition by medicinal biguanides. Science 379, 351–357, doi:10.1126/science.ade3332 (2023). 36701435 PMC7614227

[15] LaMoia T. E. & Shulman G. I. Cellular and Molecular Mechanisms of Metformin Action. Endocr Rev 42, 77–96, doi:10.1210/endrev/bnaa023 (2021). 32897388 PMC7846086

[16] Seo B. B. Molecular remedy of complex I defects: rotenone-insensitive internal NADH-quinone oxidoreductase of Saccharomyces cerevisiae mitochondria restores the NADH oxidase activity of complex I-deficient mammalian cells. Proc Natl Acad Sci U S A 95, 9167–9171, doi:10.1073/pnas.95.16.9167 (1998). 9689052 PMC21310

[17] Wheaton W. W. Metformin inhibits mitochondrial complex I of cancer cells to reduce tumorigenesis. Elife 3, e02242, doi:10.7554/eLife.02242 (2014). 24843020 PMC4017650

[18] Dowling R. J. Metformin Pharmacokinetics in Mouse Tumors: Implications for Human Therapy. Cell Metab 23, 567–568, doi:10.1016/j.cmet.2016.03.006 (2016). 27076069

[19] Izzo F. DNA methylation disruption reshapes the hematopoietic differentiation landscape. Nat Genet 52, 378–387, doi:10.1038/s41588-020-0595-4 (2020). 32203468 PMC7216752

[20] Nam A. S. Single-cell multi-omics of human clonal hematopoiesis reveals that DNMT3A R882 mutations perturb early progenitor states through selective hypomethylation. Nat Genet 54, 1514–1526, doi:10.1038/s41588-022-01179-9 (2022). 36138229 PMC10068894

[21] Dai Y. J. Conditional knockin of Dnmt3a R878H initiates acute myeloid leukemia with mTOR pathway involvement. Proc Natl Acad Sci U S A 114, 5237–5242, doi:10.1073/pnas.1703476114 (2017). 28461508 PMC5441829

[22] Fiumara M. Genotoxic effects of base and prime editing in human hematopoietic stem cells. Nat Biotechnol, doi:10.1038/s41587-023-01915-4 (2023). PMC11180610 37679541

[23] Chen P. J. & Liu D. R. Prime editing for precise and highly versatile genome manipulation. Nat Rev Genet 24, 161–177, doi:10.1038/s41576-022-00541-1 (2023). 36344749 PMC10989687

[24] SanMiguel J. M. Distinct Tumor Necrosis Factor Alpha Receptors Dictate Stem Cell Fitness versus Lineage Output in Dnmt3a-Mutant Clonal Hematopoiesis. Cancer Discov 12, 2763–2773, doi:10.1158/2159-8290.CD-22-0086 (2022). 36169447 PMC9716249

[25] Cuyas E. Metformin regulates global DNA methylation via mitochondrial one-carbon metabolism. Oncogene 37, 963–970, doi:10.1038/onc.2017.367 (2018). 29059169

[26] Cuyas E. Metformin directly targets the H3K27me3 demethylase KDM6A/UTX. Aging Cell 17, e12772, doi:10.1111/acel.12772 (2018). 29740925 PMC6052472

[27] Garcia-Calzon S. DNA methylation partially mediates antidiabetic effects of metformin on HbA1c levels in individuals with type 2 diabetes. Diabetes Res Clin Pract 202, 110807, doi:10.1016/j.diabres.2023.110807 (2023). 37356726

[28] Nelson J. W. Engineered pegRNAs improve prime editing efficiency. Nat Biotechnol 40, 402–410, doi:10.1038/s41587-021-01039-7 (2022). 34608327 PMC8930418

[29] Gehrke S. Red Blood Cell Metabolic Responses to Torpor and Arousal in the Hibernator Arctic Ground Squirrel. J Proteome Res 18, 1827–1841, doi:10.1021/acs.jproteome.9b00018 (2019). 30793910 PMC7219541

[30] Nemkov T., Reisz J. A., Gehrke S., Hansen K. C. & D’Alessandro A. High-Throughput Metabolomics: Isocratic and Gradient Mass Spectrometry-Based Methods. Methods Mol Biol 1978, 13–26, doi:10.1007/978-1-4939-9236-2_2 (2019). 31119654

[31] Nemkov T., Hansen K. C. & D’Alessandro A. A three-minute method for high-throughput quantitative metabolomics and quantitative tracing experiments of central carbon and nitrogen pathways. Rapid Commun Mass Spectrom 31, 663–673, doi:10.1002/rcm.7834 (2017). 28195377 PMC5364945

[32] Akalin A. methylKit: a comprehensive R package for the analysis of genome-wide DNA methylation profiles. Genome Biol 13, R87, doi:10.1186/gb-2012-13-10-r87 (2012). 23034086 PMC3491415

[33] Akalin A. Base-pair resolution DNA methylation sequencing reveals profoundly divergent epigenetic landscapes in acute myeloid leukemia. PLoS Genet 8, e1002781, doi:10.1371/journal.pgen.1002781 (2012). 22737091 PMC3380828

[34] Wang H. Q., Tuominen L. K. & Tsai C. J. SLIM: a sliding linear model for estimating the proportion of true null hypotheses in datasets with dependence structures. Bioinformatics 27, 225–231, doi:10.1093/bioinformatics/btq650 (2011). 21098430

[35] Lee S., Cook D. & Lawrence M. plyranges: a grammar of genomic data transformation. Genome Biol 20, 4, doi:10.1186/s13059-018-1597-8 (2019). 30609939 PMC6320618

[36] Wang Q. Exploring Epigenomic Datasets by ChIPseeker. Curr Protoc 2, e585, doi:10.1002/cpz1.585 (2022). 36286622

[37] Yu G., Wang L. G. & He Q. Y. ChIPseeker: an R/Bioconductor package for ChIP peak annotation, comparison and visualization. Bioinformatics 31, 2382–2383, doi:10.1093/bioinformatics/btv145 (2015). 25765347

