# Supplementary material for: Metformin reduces the clonal fitness of Dnmt3aR878H hematopoietic stem and progenitor cells by reversing their aberrant metabolic and epigenetic state
Source: Res Sq. 2024 Feb 6:rs.3.rs-3874821. Preprint. [Version 1] doi: 10.21203/rs.3.rs-3874821/v1 (PMC10889081; doi:10.21203/rs.3.rs-3874821/v1)
Supplement: Supplement 1 [file NIHPPrs3874821v1-supplement-1.pdf]

Figure S1

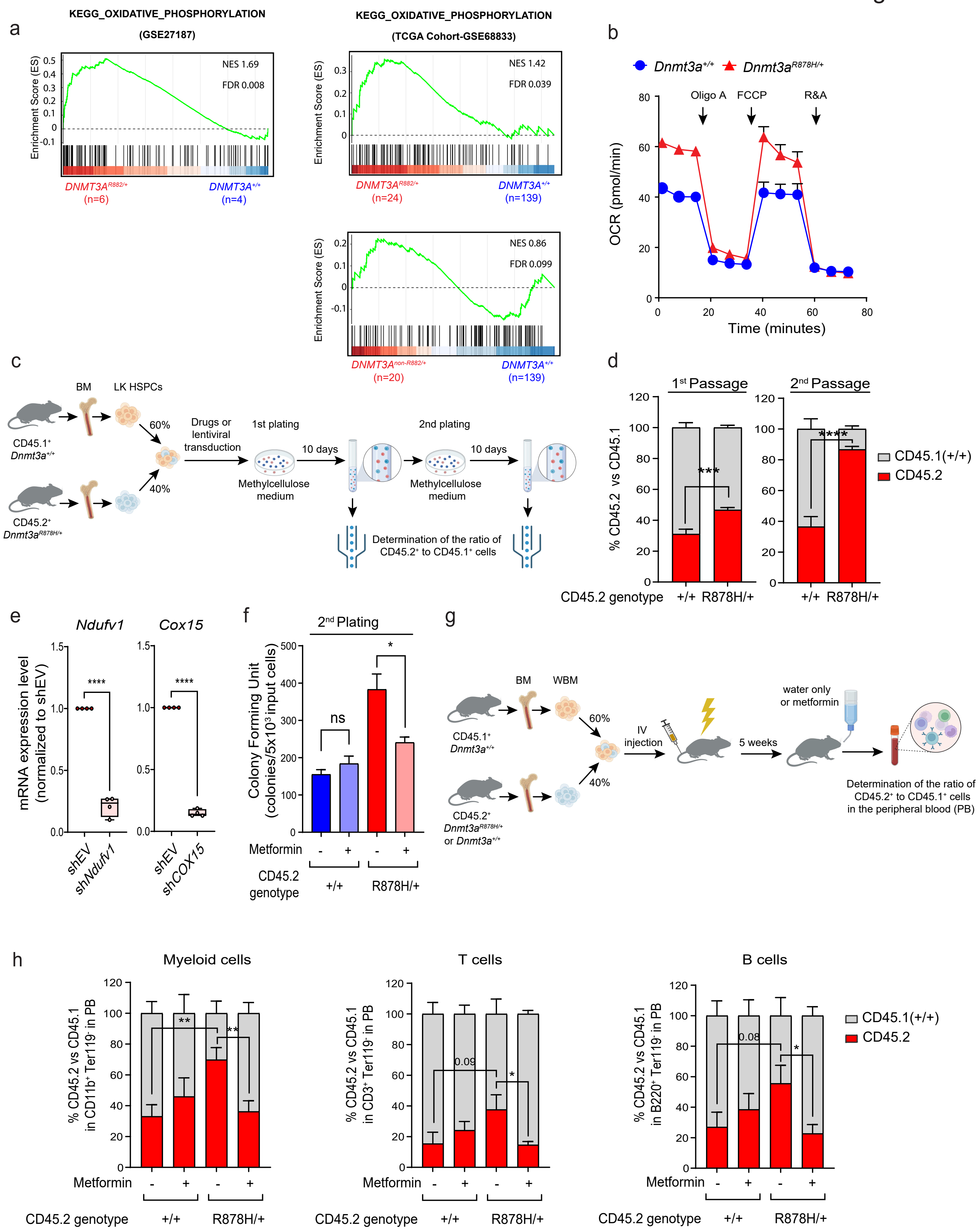

a
KEGG_OXIDATIVE_PHOSPHORYLATION
(GSE27187)
NES 1.69
FDR 0.008
Enrichment Score (ES)
DNMT3A R882/+ (n=6)
DNMT3A +/+ (n=4)
KEGG_OXIDATIVE_PHOSPHORYLATION
(TCGA Cohort-GSE68833)
NES 1.42
FDR 0.039
DNMT3A R882/+ (n=24)
DNMT3A +/+ (n=139)
NES 0.86
FDR 0.099
DNMT3A non-R882/+ (n=20)
DNMT3A +/+ (n=139)
b
Dnmt3a +/+
Dnmt3a R878H/+
Oligo A
FCCP
R&A
OCR (pmol/min)
Time (minutes)
c
BM
LK HSPCs
CD45.1+ Dnmt3a +/+
CD45.2+ Dnmt3a R878H/+
60%
40%
Drugs or lentiviral transduction
1st plating
10 days
2nd plating
10 days
Methylcellulose medium
Determination of the ratio of CD45.2+ to CD45.1+ cells
d
1st Passage
2nd Passage
% CD45.2 vs CD45.1
CD45.1(+/+)
CD45.2
***
****
CD45.2 genotype
+/+
R878H/+
e
Ndufv1
Cox15
mRNA expression level (normalized to shEV)
****
shEV
shNdufv1
shCOX15
f
2nd Plating
Colony Forming Unit (colonies/5x10³ input cells)
ns
*
Metformin
CD45.2 genotype
+/+
R878H/+
g
BM
WBM
CD45.1+ Dnmt3a +/+
CD45.2+ Dnmt3a R878H/+ or Dnmt3a +/+
60%
40%
IV injection
5 weeks
water only or metformin
Determination of the ratio of CD45.2+ to CD45.1+ cells in the peripheral blood (PB)
h
Myeloid cells
T cells
B cells
% CD45.2 vs CD45.1 in CD11b+ Ter119- in PB
% CD45.2 vs CD45.1 in CD3+ Ter119- in PB
% CD45.2 vs CD45.1 in B220+ Ter119- in PB
**
0.09
0.08
*
CD45.1(+/+)
CD45.2
Metformin
CD45.2 genotype
+/+
R878H/+

Extended Data Fig. 1 | *Dnmt3a*$^{\mathbf{R878H/+}}$ HSPCs have increased mitochondrial respiration compared with *Dnmt3a*$^{+/+}$ cells and are dependent on this metabolic reprogramming for their competitive advantage. a, Gene set enrichment plots comparing *DNMT3A*^{R882} or *DNMT3A*$^{non\text{-}R882}$ mutated AML samples versus *DNMT3A*^{WT} AML samples using two publicly available gene expression datasets (GSE27187 and GSE68833). **b,** OCRs of whole bone marrow cells of the indicated genotype at baseline and at different time points following treatment with oligomycin A (Oligo A), FCCP, and rotenone plus antimycin A (R&A). n=4-6 technical replicates for each data point. Representative data of 3 independent experiments are shown. **c,** Schematic diagram showing the design of the *in vitro* competition assay. **d,** Proportion of CD45.2$^+$ and CD45.1$^+$ cells in a competition assay between CD45.2$^+$ LK cells of the indicated genotype and CD45.1$^+$ *Dnmt3a*$^{+/+}$ LK cells after the 1st passage (n=8 technical replicates) and 2nd passage (n=13 technical replicates). **e,** Expression of the indicated genes in *Dnmt3a*$^{+/+}$ LK cells transduced with an empty shRNA vector control (shEV) or a shRNA vector expressing sh*Ndufv1* or sh*Cox15*. Dots represent technical replicates. **f,** Number of colony forming units in the second plating from *Dnmt3a*$^{+/+}$ or *Dnmt3a*$^{R878H/+}$ LK HSPCs in the absence or presence of metformin. n=3 technical replicates per condition. Representative data of 3 independent experiments are shown. **g,** Schematic diagram showing the design of the *in vivo* competitive repopulation experiment. **h,** Proportion of CD45.2$^+$ vs. CD45.1$^+$ cells in the myeloid (CD11b$^+$), T (CD3$^+$), and B (B220$^+$) cell compartments in peripheral blood cells collected from mice after 4 months of treatment with metformin or vehicle in the experiment shown in Fig. 1h. n=7 mice per condition. In **e,** the box represents the interquartile range with the median indicated by the line inside the box. Whiskers extend to the minimum and maximum values. In **b, d, f, h,** data shown are mean ± SEM. Statistical significance (P values) were calculated using two-sided Student's t-test for all comparisons. * P<0.05, ** P<0.01, *** P< 0.001, and *** P<0.0001. ns, not significant.

Figure S2

a

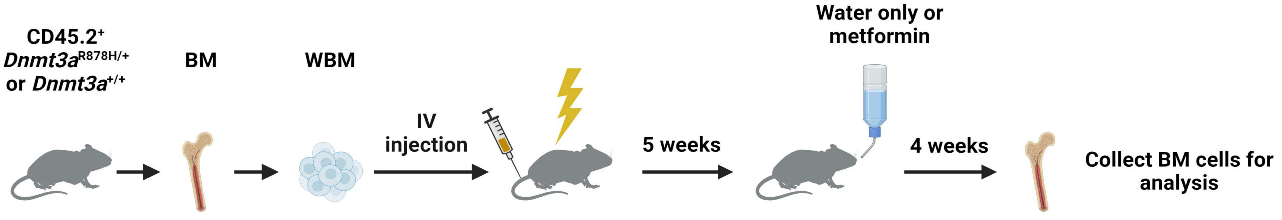

CD45.2+
Dnmt3aR878H/+
or Dnmt3a+/+
BM
WBM
IV
injection
5 weeks
Water only or
metformin
4 weeks
Collect BM cells for
analysis

Extended Data Fig. 2 | Metformin suppresses the competitive advantage of *Dnmt3a*$^{R878H/+}$ HSCs. a, Schematic diagram showing the design of the non-competitive repopulation experiment in which lethally-irradiated recipient mice were transplanted with CD45.2^{+} *Dnmt3a*$^{+/+}$ or *Dnmt3a*$^{R878H/+}$ whole bone marrow (WBM) cells. Five weeks after transplantation, the mice were either left untreated or treated with metformin in the drinking water. After 4 weeks of treatment, BM cells were collected and used for analysis.

Figure S3

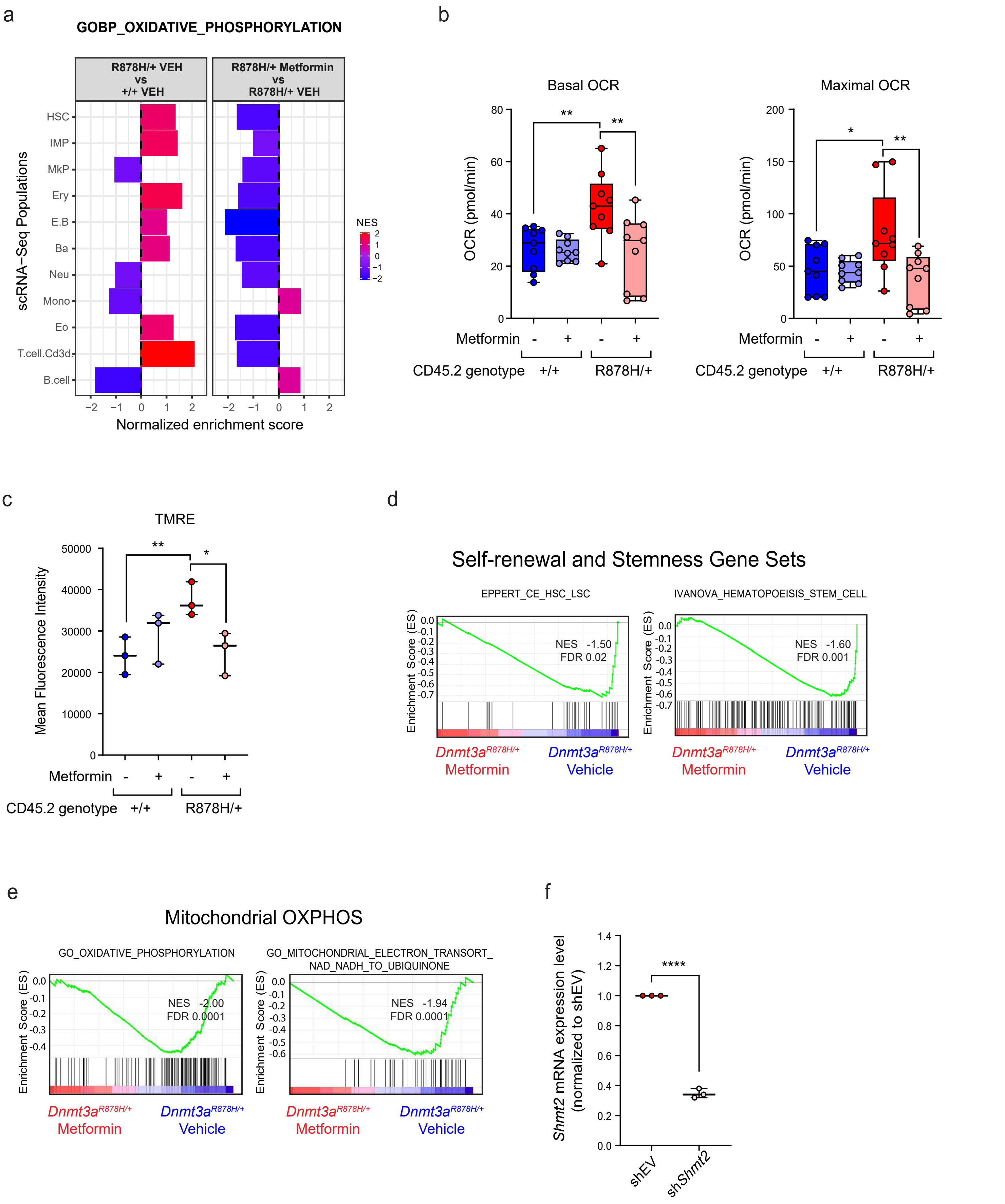
a
GOBP_OXIDATIVE_PHOSPHORYLATION
R878H/+ VEH vs +/+ VEH
R878H/+ Metformin vs R878H/+ VEH
scRNA-Seq Populations
HSC
IMP
MkP
Ery
E.B
Ba
Neu
Mono
Eo
T.cell.Cd3d
B.cell
NES
Normalized enrichment score
b
Basal OCR
Maximal OCR
OCR (pmol/min)
Metformin - + - +
CD45.2 genotype +/+ R878H/+
c
TMRE
Mean Fluorescence Intensity
Metformin - + - +
CD45.2 genotype +/+ R878H/+
d
Self-renewal and Stemness Gene Sets
EPPERT_CE_HSC_LSC
NES -1.50
FDR 0.02
IVANOVA_HEMATOPOEISIS_STEM_CELL
NES -1.60
FDR 0.001
Enrichment Score (ES)
Dnmt3a R878H/+ Metformin
Dnmt3a R878H/+ Vehicle
e
Mitochondrial OXPHOS
GO_OXIDATIVE_PHOSPHORYLATION
NES -2.00
FDR 0.0001
GO_MITOCHONDRIAL_ELECTRON_TRANSORT_ NAD_NADH_TO_UBIQUINONE
NES -1.94
FDR 0.0001
Enrichment Score (ES)
Dnmt3a R878H/+ Metformin
Dnmt3a R878H/+ Vehicle
f
Shmt2 mRNA expression level (normalized to shEV)
****
shEV
shShmt2

Extended Data Fig. 3 | Metformin suppresses the fitness of *Dnmt3a*$^{R878H/+}$ HSPCs by enhancing their methylation potential. a, Normalized enrichment scores (NES) for the indicated gene set (GO: 0006119) in each HSPC subset using scRNA-seq gene expression data. **b,** Basal and maximal OCRs in LK HSPCs collected from mice transplanted with WBM cells of the indicated genotype and treated with or without metformin for 1 month. Dots represent samples from individual mice. **c,** Mean fluorescence intensity of TMRE staining in LK HSPCs collected from mice transplanted with WBM cells of the indicated genotype and treated with or without metformin for 1 month. Dots represent samples from individual mice. **d.** Gene set enrichment plots of bulk RNA-seq data comparing metformin-treated *Dnmt3a*$^{R878H/+}$ LK cells (n=2 biological replicates) versus vehicle-treated *Dnmt3a*$^{R878H/+}$ LK cells (n=2 biological replicates) using the indicated self-renewal and stemness-related gene sets. **e.** Gene set enrichment plots of bulk RNA-seq data comparing metformin-treated *Dnmt3a*$^{R878H/+}$ LK cells (n=2 biological replicates) versus vehicle-treated *Dnmt3a*$^{R878H/+}$ LK cells (n=2 biological replicates) using the indicated mitochondrial function-related gene sets. **f,** Expression of *Shmt2* in *Dnmt3a*$^{+/+}$ LK cells transduced with an empty shRNA vector control (shEV) or a shRNA vector expressing sh*Shmt2*. n=3 technical replicates per condition. In **b, c, f**, the box represents the interquartile range with the median indicated by the line inside the box. Whiskers extend to the minimum and maximum values. Statistical significance (P values) was calculated using two-sided Student's t-test for all comparisons. * $P<0.05$, ** $P<0.01$, *** $P< 0.001$, and *** $P<0.0001$.

Figure S4

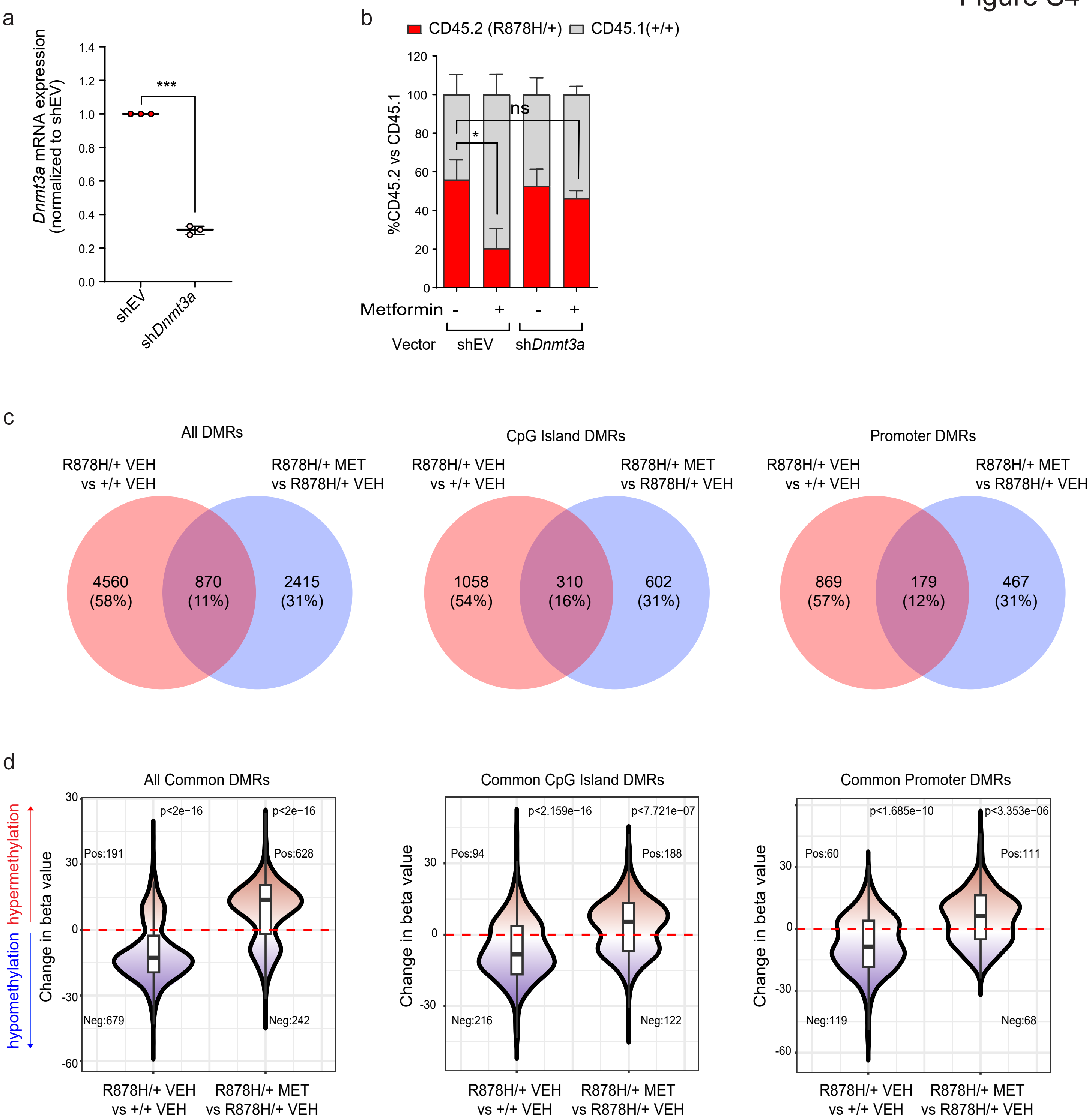
a
Dnmt3a mRNA expression (normalized to shEV)
***
shEV
shDnmt3a
b
CD45.2 (R878H/+)
CD45.1(+/+)
%CD45.2 vs CD45.1
ns
*
Metformin
-
+
-
+
Vector
shEV
shDnmt3a
c
All DMRs
R878H/+ VEH vs +/+ VEH
R878H/+ MET vs R878H/+ VEH
4560 (58%)
870 (11%)
2415 (31%)
CpG Island DMRs
R878H/+ VEH vs +/+ VEH
R878H/+ MET vs R878H/+ VEH
1058 (54%)
310 (16%)
602 (31%)
Promoter DMRs
R878H/+ VEH vs +/+ VEH
R878H/+ MET vs R878H/+ VEH
869 (57%)
179 (12%)
467 (31%)
d
All Common DMRs
hypomethylation
hypermethylation
Change in beta value
p<2e−16
p<2e−16
Pos:191
Pos:628
Neg:679
Neg:242
R878H/+ VEH vs +/+ VEH
R878H/+ MET vs R878H/+ VEH
Common CpG Island DMRs
Change in beta value
p<2.159e−16
p<7.721e−07
Pos:94
Pos:188
Neg:216
Neg:122
R878H/+ VEH vs +/+ VEH
R878H/+ MET vs R878H/+ VEH
Common Promoter DMRs
Change in beta value
p<1.685e−10
p<3.353e−06
Pos:60
Pos:111
Neg:119
Neg:68
R878H/+ VEH vs +/+ VEH
R878H/+ MET vs R878H/+ VEH

Extended Data Fig. 4 | Metformin reverses the aberrant DNA CpG methylation and H3K27me3 profiles in *Dnmt3a*$^{R878H/+}$ HSPCs. a, Expression of *Dnmt3a* in *Dnmt3a*$^{+/+}$ LK cells transduced with an empty shRNA vector control (shEV) or a shRNA vector expressing sh*Dnmt3a*. n=3 technical replicates per condition. **b,** Proportion of CD45.2^{+} and CD45.1^{+} cells in a competition assay between CD45.2^{+} *Dnmt3a*$^{R878H/+}$ LK cells and CD45.1^{+} *Dnmt3a*$^{+/+}$ LK cells in the presence or absence of metformin. Both populations were transduced with the indicated shRNA vectors. n=4-6 technical replicates. Representation data of 3 independent experiments are shown. **c,** Venn diagram showing the overlap of DMRs between metformin-treated *Dnmt3a*$^{R878H/+}$ samples versus untreated *Dnmt3a*$^{R878H/+}$ samples and between untreated *Dnmt3a*$^{R878H/+}$ samples versus untreated *Dnmt3a*$^{+/+}$ samples. **d,** Violin plots of the difference in beta values at the overlapping DMRs in the comparison between untreated *Dnmt3a*$^{R878H/+}$ LK samples versus untreated *Dnmt3a*$^{+/+}$ LK samples (left) and between metformin-treated *Dnmt3a*$^{R878H/+}$ LK samples versus untreated *Dnmt3a*$^{R878H/+}$ LK samples (right). The P values adjacent to the plots were calculated using the one-sample Wilcoxon signed rank test to determine if the median difference in beta values was significantly different from 0. In **a,** the box represents the interquartile range with the median indicated by the line inside the box. Whiskers extend to the minimum and maximum values. In **b,** data shown are mean ± SEM. Statistical significance (P values) were calculated using two-sided Student's t-test for all comparisons. * P<0.05, ** P<0.01, *** P< 0.001, and *** P<0.0001. ns, not significant.

Extended Table 1 | Metabolite levels in metabolomics analysis

Extended Table 2 | List of RNA sequences used for prime editing

Extended Table 3 | List of primer and oligonucleotides sequences used in the study

Extended Table 4 | List of antibodies used in the study

Extended Table 5 | List of chemicals and reagents used in the study
